# siRNA Interaction and Transfection Properties of Polycationic Phosphorus Dendrimers

**DOI:** 10.1021/acs.biomac.5c00171

**Published:** 2025-05-30

**Authors:** Irene Rodríguez-Clemente, Andrii Karpus, Angel Buendía, Krzystof Sztandera, Elzbieta Regulska, Jerome Bignon, Anne-Marie Caminade, Carlos Romero-Nieto, Anke Steinmetz, Serge Mignani, Jean-Pierre Majoral, Valentín Ceña

**Affiliations:** † Unidad Asociada Neurodeath, INAMOL, 16733 Universidad de Castilla-La Mancha, Albacete 02006, Spain; ‡ CIBER, Instituto de Salud Carlos III, Madrid 28028, Spain; § Department of Inorganic, Organic and Biochemistry, University of Castilla-La Mancha, Albacete 02006, Spain; ∥ IRICA, Universidad de Castilla-La Mancha, Ciudad Real 13071, Spain; As ⊥ Laboratoire de Chimie de Coordination, CNRS, Toulouse Cedex 4 31077, France; # Sanofi R&D, CMC Synthetics/BTDV/EB, CRV, Vitry-sur-Seine 94400, France; ∇ Centre d’Etudes et de Recherche sur le Medicament de Normandie (CERMN), Université de Caen Normandie, Caen 14032, France; ○ CQM—Centro de Química da Madeira, MMRG, Universidade da Madeira, Funchal 9000·390, Portugal

## Abstract

Glioblastoma multiforme (GBM) is the most common type of primary brain tumor in adults and has a poor prognosis. Small interfering RNA (siRNA) can disrupt different mechanisms involved in the genesis of several diseases including GBM. However, siRNA complexation with nonviral cationic carriers is required to transport siRNAs inside cells and promote its function. We have designed and synthesized new cationic phosphorus dendrimers bearing either 6, 12, or 24 pyrrolidinium or piperidinium groups on their surface. These dendrimers bound siRNA, those bearing terminal pyrrolidinium having the highest affinity. However, they showed marked differences in protecting siRNA from RNase-mediated degradation. Molecular modeling suggested that, beyond the overall protonation status, the intrinsic flexibility and individual binding properties of these dendrimers contributed to the modulation of biological profiles. However, these phosphorus dendrimers were unable to transport significant amounts of siRNA into GBM cells and, accordingly, transfection was inefficient.

## Graphical abstract

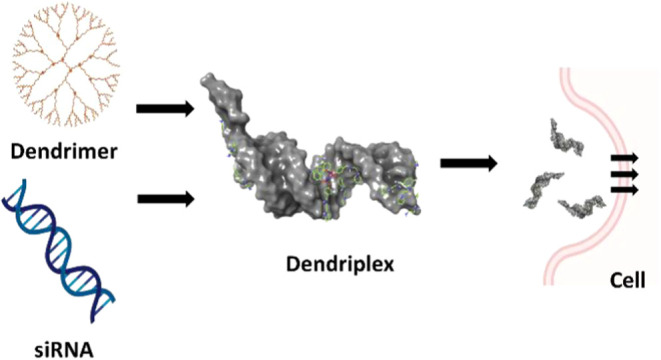

## Introduction

Glioblastomas (GBMs) are the most common type of primary brain tumor in adults.^1^ The standard treatment for GBM includes maximal surgical resection followed by radiotherapy and concomitant Temozolomide (TMZ)-based maintenance chemotherapy.^2^ Despite the standard treatments administered, median patient survival is about 14 months from the time of diagnosis, and, after recurrence, median survival is about 6 months. Thus, the overall patient survival rate at 2 years from the time of the primary diagnosis is about 27%.^3^ Hence, research on new treatments is required to further improve survival in patients with GBM.

Interference RNA (RNAi) is a physiological mechanism that operates in most eukaryotic cells^4^ to either inhibit transcription or induce sequence-specific mRNA degradation. Thus, RNAis are involved in the regulation of cellular metabolism, replication, and malignant transformation.^5^ Small interfering RNA (siRNA) is a synthetic double-stranded compound comprising 20 to 24 base pairs that mimics the endogenous RNAi system to interfere with different mechanisms involved in the genesis of several diseases. These molecules are highly specific and can rapidly and markedly decrease target protein levels.^6^ Moreover, several siRNA-based drugs have already been approved by the United States Food and Drug Administration (FDA).^7^

However, siRNAs have several drawbacks including their low stability, negative surface polarization, and susceptibility to enzymatic degradation which all limit the efficiency of their delivery into the target cells and use as therapeutic agents. Thus, complexation with nonviral cationic carriers is required to facilitate their transport into cells and subsequent functioning. Nonviral vectors have several advantages over viral vectors, including the simplicity of their preparation, stability, biosafety, and the fact that their surfaces are tunable. This type of vector includes inorganic particles, lipids, and different polymer types, as well as dendritic species. Furthermore, among these vector families, dendrimers can play an important role in the protection and delivery of siRNAs. This is because they have a uniform shape and size, their backbones contain free voids, and their surfaces present numerous tunable functional groups.^8^

Among the many types of dendrimers available, phosphorus dendrimers have demonstrated activity as antiprion,^9^ anti-HIV,^10^ antituberculosis,^11^ anti-inflammatory,^12^ neuroprotective agents,^13^ in chemotherapy of tumors,^14^ and potentially, also in the treatment of Parkinson disease^15^ because of their hydrophilic surface and hydrophobic backbone. Polycationic phosphorus dendrimers represent a subset of this dendrimer family with high potential as siRNA carriers resulting from their multivalency and water solubility. Indeed, cationic phosphorus dendrimers have also been used as promising gene-delivery platforms. Their most widely studied species bear a cyclotriphosphazene core and diethyl amines at their branch termini.^16^

The vast majority of phosphorus dendrimer syntheses to date have involved several reactions using methylhydrazine, leading to the formation of water-sensitive internal and/or terminal CH=N–N­(CH_3_)–P units. These families of phosphorus dendrimers, prepared up to generation 12, have been extensively used for many applications despite their sensitivity to hydrolysis. Therefore, efforts have been directed not only toward the preparation of stable, original phosphorus dendrimers but also toward the synthesis of low-generation phosphorus dendrimers, which offer several key advantages: few steps (3–4) required to reach generation 0–2 with high yields; high water solubility; stability for over two years; suitability for preparation under GMP conditions in large quantities; ease of modifying the nature and number of terminal groups; and inexpensive starting materials.

In this current work, considering the high potential of polycationic phosphorus dendrimers to form dendriplexes with siRNAs, we were interested in designing and using the above indicated new types of these dendritic macromolecules to efficiently transfect GBM cells. We (S. Mignani and J.P. Majoral) designed and synthesized original cationic phosphorus dendrimers of different generations and with varying structures, bearing either 6, 12, or 24 pyrrolidinium or piperidinium groups on their surface, thus allowing us to gain insights into their structure–activity relationship. We characterized their interaction with siRNA and the protection they conferred against RNase-mediated siRNA degradation. In addition, we tested the toxicity of these compounds on several tumor cell lines as well as primary astrocyte cultures. Further, we studied the ability of all these compounds to deliver siRNA into cell interiors and the transfection efficiency of the cationic phosphorus dendrimers.

## Experimental Section

### Phosphorus Dendrimer Synthesis

The strategy developed in this work aimed to simplify the synthesis of perfectly stable, low generation, polycationic phosphorus dendrimers that are soluble in water, and easily prepared at a gram-scale (Scheme 1). These dendrimers bear from 12 to 24 terminal pyrrolidinium or piperidinium groups and differ in both their internal and/or external structures. All these phosphorus dendrimers were perfectly stable for more than two years and were prepared in high-yield conditions.

**Scheme 1:**
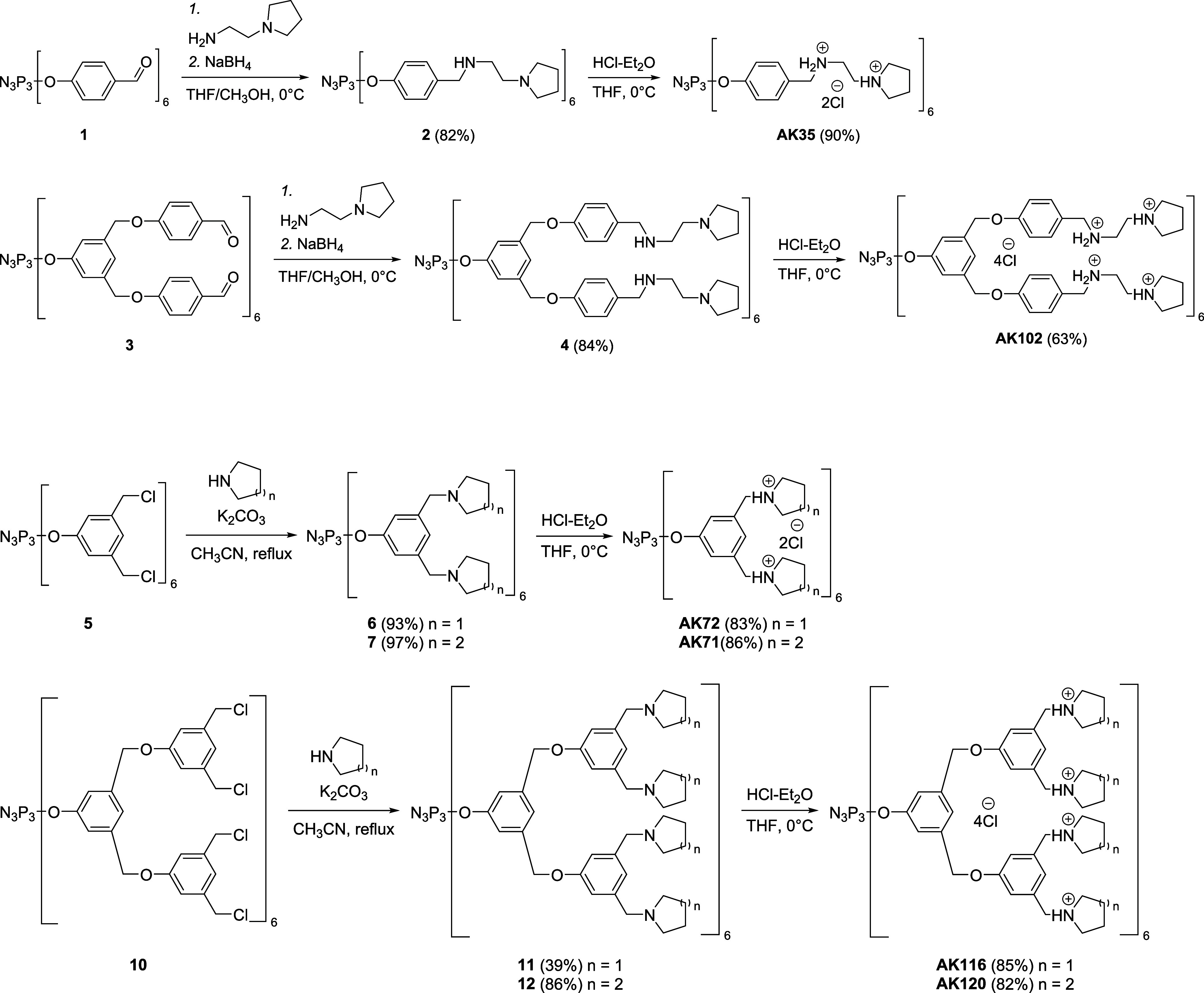
Synthesis of the Cationic Phosphorus Dendrimers AK35, AK102, AK71, AK72, AK116, and AK120

Indeed, for these experiments, three different types of phosphorus dendrimers were synthesized:

(a) Phosphorus dendrimers AK35 and AK102 incorporate terminal groups on their surface: six protonated amino groups and six pyrrolidinium units for AK35; 12 protonated amino groups and 12 pyrrolidinium groups for AK102.
(b) Phosphorus dendrimers AK71 and AK72 incorporate either 12 pyrrolidinium groups (AK72) or 12 piperidinium groups (AK71).
(c) Phosphorus dendrimers AK116 and AK120 incorporate, respectively, 24 pyrrolidinium groups (AK116) or 24 piperidinium groups (AK120).

### Molecular Modeling

Molecular modeling was performed using a graphical interface and Small Molecule Drug Discovery Suite software released in 2020 or later (Schrödinger Inc., New York), unless indicated otherwise. Computational exploration and results analysis were conducted for the AK35 phosphorus dendrimer, as previously described in detail.^11^ More specifically, conformational searches and molecular dynamics (MD) simulations were conducted on three different protonation states of AK35: all secondary amines either protonated or deprotonated, or the secondary amines of two branches above and one branch below the plane of the N3P3 cycle as protonated and the remaining secondary amines as deprotonated; tertiary amines were always protonated. In addition, models of AK71 and AK72 were also constructed following the same procedure and were subjected to conformational searches to obtain low-energy conformations. AK71 and AK72 were modeled with all tertiary amines protonated.

An all-atom three-dimensional model of siRNAs with 5′→3′ sense UACUCAGAUCGUGUCACGUdTdT and 3′→5′ antisense dTdTAUGAGUCUAGCACAGUGCA sequences was constructed by Web server Vfold3D.^17^ The resulting model was compared to the siRNA from Protein Data Bank (PDB) entries 1R9F and 2F8S, or the 17-mer RNA duplex of 4KYY.^18–20^ It fits well with these experimentally determined models in terms of helical diameter, rotational angle, and translational distance along the central axis. To prepare it for docking studies it was subjected to MD simulations after neutralizing the system at pH 7 with potassium ions (K^+^), constructing an explicit simple point-charge (SPC) water model, including 0.154 M potassium chloride (KCl), and standard pre-equilibration.^21^ Four RNA conformations most diverse at the 5′ and 3′ ends were identified by clustering while the central regions of the models were very similar. The models were centered at the origin and the helical axes were aligned with axis z. Five grids for docking were calculated on model 1, centered at the origin or shifted along axis *z* to the ±20 and ±40 Å positions. Six additional grids were calculated on models 2 to 4, at *z* positions ±40 Å to capture the conformational diversity at the 5′ and 3′ ends for interactions with dendrimers. Docking was performed with the XGlide cross-docking script with standard settings by providing the precalculated grids, AK35 models of all three protonation states, and fully protonated models of AK71 and AK72, all in the lowest energy conformation issued from conformational searches.^22^ Furthermore, an AK35/siRNA dendriplex with stoichiometry 13:1 was stepwise constructed by docking and subjected to two MD simulations of 1 μs under standard conditions. For details, see the Supporting Information.

### Cell Culture

Cell lines were obtained from the American Type Culture Collection (Rockville, MD) and were cultured according to the supplier’s instructions. Briefly, human HCT-116 colorectal carcinoma and U2OS osteosarcoma cells were grown in Gibco McCoy’s 5A supplemented with 10% fetal calf serum (FCS) and 1% glutamine. MDA-MB231 breast carcinoma, A549 lung carcinoma, and K562 leukemia cells were grown in RPMI 1640 supplemented with 10% fetal calf serum and 1% glutamine. U87-MG glioblastoma cells, T98G human glioblastoma cells, and the human MRC-5 cell line derived from normal lung tissue were grown in Dulbecco’s minimal essential medium (DMEM; Thermo Fisher; Waltham, MA) containing 4.5 g/L glucose supplemented with 10% FCS and 1% glutamine. Human hTERT-RPE1 cells were cultured in DMEM/F12 medium containing 10% FCS and 1% glutamine. All cell lines were maintained at 37 °C in a humidified atmosphere containing 5% CO_2_.

Primary astrocytes were isolated from one-day-old mouse pups and cultured in DMEM supplemented with 10% FCS, 2 mM l-glutamine, 100 μg/mL streptomycin, and 100 IU/mL penicillin in a saturated humidified atmosphere containing 95% air and 5% CO_2_ maintained at 37 °C as previously described.^23^ The animal experimental study was approved by the Animal Experimentation Ethics Committee at the University of Castilla-La Mancha (UCLM; protocol number PR-2014–10–12) and carried out in accordance with the guidelines from the same UCLM committee and the European Union (directive 2010/63/EU) for the use of laboratory animals.

### Gel Retardation

Assays were performed as previously described^24^ with minor modifications. Phosphorus dendrimer/siRNA complexes were prepared at increasing protonable nitrogen/phosphorus (N/P) ratios by incubation at room temperature for 30 min. Dendriplexes were then loaded onto a 1.2% agarose gel containing 0.004% (v/v) Red Safe (Intron Biotech, South Korea) in TAE buffer (40 mM Tris base, 20 mM glacial acetic acid, and 1 mM ethylenediamine tetra-acetic acid [EDTA], at pH 8.6). Samples were electrophoresed (60 V for 20 min) and photographed under UV-illumination. Gel band intensities were measured using ImageJ software.^25^

### RNA Protection against RNases

Phosphorus dendrimers-mediated protection against siRNA degradation by ribonuclease A (RNase) was performed as previously described.^26^ Briefly, nanoparticle (NP)–siRNA complexes were prepared as indicated above. Complexes, or naked siRNA (100 nM), were incubated in the presence of 0.25% RNase A (Sigma, Barcelona, Spain) for 30 min at 37 °C. The sample was then cooled at 4 °C for 20 min to inactivate the RNase and incubated with heparin (1.5 IUs) at 4 °C for additional 20 min to release the siRNA from the phosphorus dendrimer. Next, the samples were processed, and the sample bands were quantified as described above.

### Circular Dichroism

Circular dichroism (CD) spectra were recorded, in the absence and presence of siRNA, using an OLIS DSM 170 CD spectrophotometer equipped with a 150 W xenon lamp (OLIS, Athens, GA). Data were collected with a 2 nm bandwidth.

### Hydrodynamic Radius and Zeta (ζ) Potential

The hydrodynamic diameter of particles was measured using dynamic light scattering (DLS) with the Malvern Instrument Zetasizer Nano ZS (Malvern Instruments, Worcerstershire, U.K.). The scattered light was collected using noninvasive back scatter technology at 173° angle. The zeta potential was determined with the same instrument using the electrophoretic light scattering (ELS) method. The scattered light was collected at 13° forward scattering angle.

### Toxicity

#### Proliferation Assay

Cell viability for all the cell types but the T98G and astrocytes was determined by a luminescent assay according to the manufacturer’s instructions (Promega, Madison, WI). To determine the IC_50_, the cells were seeded in 96-well plates (3 × 103 cells/well) containing 90 μL of growth medium. After 24 h of culture, the cells were treated with the tested compounds at 10 different final concentrations. Each concentration was obtained from serial dilutions in culture medium starting from the stock solution. Control cells were treated with the vehicle. The experiments were performed in triplicate. After 72 h of incubation, 100 μL of CellTiter Glo Reagent was added for 15 min before recording the luminescence with a PolarStar Omega (BMG LabTech) spectrophotometric plate reader. The dose–response curves were plotted and the IC_50_ values were calculated from polynomial curves (four or five-parameter logistic equations) using GraphPad Prism software (GraphPad Software; Boston).

#### Lactate Dehydrogenase Release

Toxicity in T98G and astrocytes was measured by determining the release of lactate dehydrogenase (LDH) to the culture medium using the CytoTox96 Non-Radioactive Cytotoxicity Assay kit (Promega, Madrid, Spain), as previously described.^27^

Briefly, cells were incubated for 72 h with increasing concentrations of phosphorus dendrimers ranging from 1 nM to 10 μM. The culture media were then collected, the cells lysed using 0.1% (w/v) Triton X-100 in NaCl (0.9%), and the LDH content in both the culture media and cell lysates were determined using a spectrophotometer (Infinite 200, Tecan, Salzburg, Austria). LDH release was calculated as the LDH released/total LDH ratio, with the latter being the sum of the LDH content in the culture medium plus the cellular LDH content.

#### Hemolysis Assay

Blood compatibility of phosphorus dendrimers was confirmed by hemolysis assay as previously described.^28^ Blood was collected from male Swiss mice (Janvier) using 25G syringes (BD Microlance) and centrifuged to isolate red blood cells (RBCs) following the protocol number PR-2023–06 approved by the Committee of Ethics and Animal Experimentation of the University of Castilla-La Mancha on April 28, 2023. RBCs were washed 3 times with PBS 1× pH 7.4 and resuspended in PBS 1× pH 7.4. 1% RBC suspension was incubated with rising concentrations of phosphorus dendrimers (0.1, 0.5, 1, 5, and 10 μM) at 37 °C for 3h in agitation. Positive control was prepared with 1% RBC suspension in 1% Trito (Sigma, Barcelona Spain)- PBS. After the incubation, samples were centrifugated and supernatants were loaded into a 96-well plate. The absorbance was measured at 540 nm using an Infinite200 Tecan spectrophotometer. The percentage of hemolysis was calculated

$$\%\mathrm{hemolysis}=\frac{\mathrm{sample}\;\mathrm{absorbance}-\mathrm{blank}\;\mathrm{absorbance}}{\mathrm{positive}\;\mathrm{control}-\mathrm{blank}\;\mathrm{absorbance}}$$

### Cellular Uptake of Small Interfering RNA

siRNA cellular uptake was studied as previously described.^29^ Briefly, cells were seeded on 20 mm glass coverslips placed in 6-well plates in DMEM medium containing 10% FCS (Gibco, Whatman, MA), antibiotics (penicillin, 100 IU/mL and streptomycin, 100 μg/mL), and l-glutamine (2 mM), and were allowed to attach to the coverslips. Dendriplexes were prepared by incubating the different phosphorus dendrimers (AK35, 5 μM; AK71, 5 μM; AK72, 0.5 μM; AK102, 5 μM; AK116, 0.5 μM; and AK120, 0.5 μM) with 5′-carboxyfluorescein (FAM)-labeled siRNA (100 nM; Merck, KGaA, Darmstadt, Germany) for 1 h at room temperature in DMEM. Next, 10% FCS, antibiotics, and l-glutamine were added to generate complete medium. The cell culture incubation medium was then replaced with medium containing the dendriplexes.

Ten minutes before recording the data, Hoechst 33342 (25 μg/mL; ThermoFisher, Waltham, MA) was added to the culture medium. After 6 h of incubation, the cells were washed 3 times with Krebs–Henseleit (KH) solution (NaCl 140 mM, CaCl_2_ 2.5 mM, MgCl_2_ 1 mM, KCl 5 mM, *N*-2-hydroxyethylpiperazine-*N*′-2-ethanesulfonic acid [HEPES] 5 mM, and glucose 11 mM, at pH 7.4) and were then mounted in a recording chamber on the stage of a Nikon Eclipse TE2000-E fluorescence microscope (Nikon, Tokyo, Japan). Images were recorded using a 60× fluorescence, oil immersion objective and an ORCA camera (Hamamatsu, Hamamatsu City, Japan) and were analyzed using NIS Elements AR software (Nikon, Tokyo, Japan). The excitation and emission wavelengths were set at 488 and 520 nm for FAM-siRNA and at 350 and 450 nm for Hoechst 33342, respectively. Intracellular fluorescence intensity quantification was performed using ImageJ software^25^ and the data analysis was conducted using GraphPad Prism software.

### Transfection Studies

Western blot analysis was performed as previously described.^26,30^ Briefly, cells were treated for 72 h with the nanocomplexes formed by the indicated phosphorus dendrimers (AK35, 5 μM; AK71, 5 μM; AK72, 0.5 μM; AK102, 5 μM; AK116, 0.5 μM; and AK120, 0.5 μM) or the β-cyclodextrin derivative AMC11 (1 μM)^6^ and the specific siRNA (25 to 100 nM) targeting p42-mitogen-activated protein kinase (p42-MAPK; positions 355–377 of the mRNA sequence with access code NM_138957.2 in the National Center for Biotechnology Information [NCBI] nucleotide database). The cells were then washed 3 times using KH solution, lysed, and the protein content was determined by the bicinchoninic acid (BCA) protein assay (Pierce Biotechnology, Rockford, IL).

Protein samples containing 30 μg of protein were loaded onto 12% sodium dodecyl sulfate polyacrylamide gel electrophoresis (SDS-PAGE) gels and run at 90 V. The gels were then transferred to nitrocellulose membranes (Bio-Rad Laboratories, Madrid, Spain). The membranes were blocked in phosphate-buffered saline (PBS)-Tween 20 containing 5% nonfat dry milk and 0.1% bovine serum albumin (BSA) protein for 2 h at 4 °C and were then incubated with the corresponding primary antibody overnight at 4 °C. Immunoreactive bands were visualized using an enhanced chemiluminescence (ECL) system (Millipore, Madrid, Spain). Glyceraldehyde 3-phosphate dehydrogenase (GAPDH) was used as loading control. The immunoblot analysis was performed using the following primary antibodies: polyclonal anti-p42-MAPK (Erk2) antibody (1:500; Cell Signaling Technology, Leiden, The Netherlands) and monoclonal anti-GAPDH antibody (1:2000; Cell Signaling Technology, Leiden, The Netherlands). Immunoreactive bands were quantified by densitometric analysis using ImageJ software^25^ and the results were expressed as the ratio of the targeted protein density (p42-MAPK)/density of GAPDH, using the latter as a loading-control protein.

## Results and Discussion

### Interaction of Phosphorus Dendrimers with siRNA

Small interfering RNA has emerged as a new therapeutic tool for treating different diseases, many of which still lack an effective treatment. To date, several siRNA-based drugs have been approved by the United States FDA including: patisiran,^31^ givosiran,^32^ lumasiran,^33^ inclisiran,^34^ and vutrisiran^35^ while many others are in different phases of clinical trials.^7^ A critical research area in nanomedicine concerns the use of a carrier system as a vector to efficiently deliver nonviral siRNA into the brain in order to fight different diseases. In this sense, many different types of vectors have been used, including inorganic particles, lipids, or different types of polymers, as well as dendritic species. Indeed, a β-cyclodextrin derivative^6^ and polyamidoamine (PAMAM) dendrimers encapsulating gold nanoparticles were recently identified as compatible carriers to deliver therapeutic siRNA to glioma cells.^36^ In the present work, we aimed at simplifying the synthesis of the nanoparticles used as siRNA carriers by using only low generation, perfectly stable, polycationic phosphorus dendrimers soluble in water and easily prepared at gram scale (Scheme 1). These phosphorus dendrimers bear 6 to 24 terminal pyrrolidinium or piperidinium groups and differ in both their internal or external structures. Moreover, they were perfectly stable for more than two years and prepared in high yield. All the cationic phosphorus dendrimers were able to bind siRNA (Figure 1), as previously described for cationic NPs.^26^ However, they bound the siRNA with different affinity: AK35, AK71, were able to completely bind 100 nM siRNA at 5 μM while AK102 completely bound 100 nM siRNA at 1 μM AK72, AK116 and AK120 were more potent and completely bound 100 nM siRNA at 0.5 μM (Figure 1): AK35 (6 pyrrolidinium end groups) and AK71 (12 piperidinium end groups) showed the lowest affinity and thus, required a 10-fold higher concentration to completely bind 100 nM siRNA compared to the other phosphorus dendrimers.

**Figure 1:**
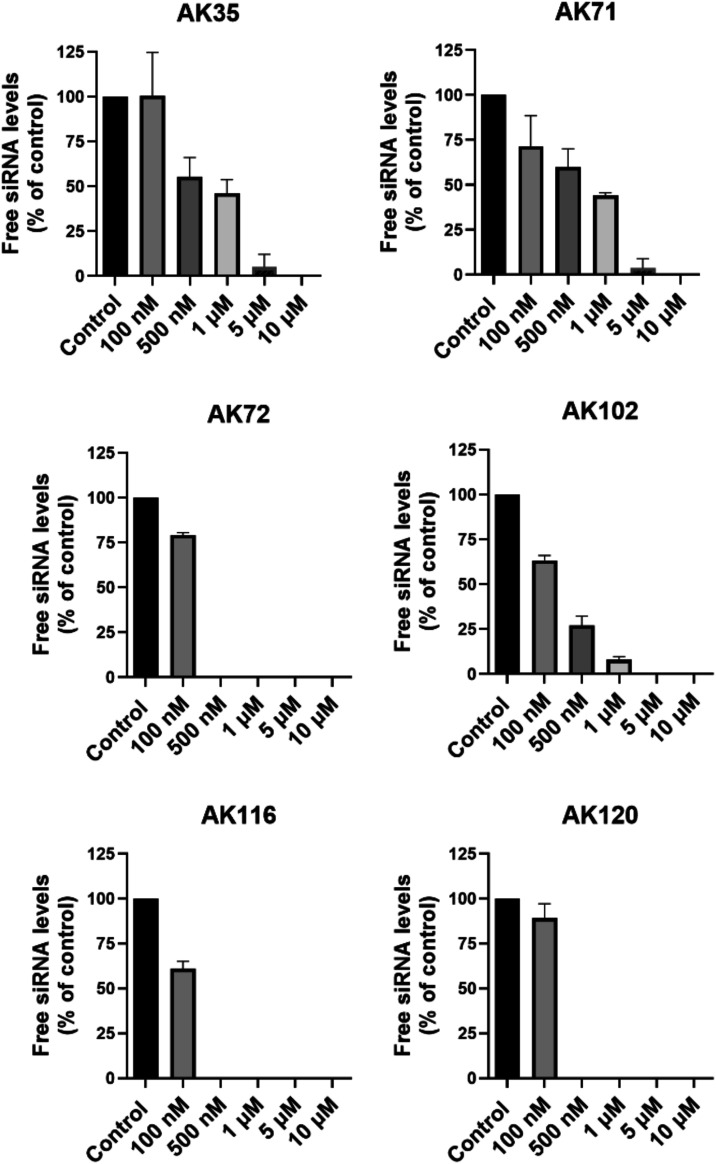
Gel retardation. siRNA (100 nM) was incubated with increasing concentrations of phosphorus dendrimers and the free siRNA quantified as indicated in Methods. Data represent mean ± s.e.m of 3 experiments.

Interestingly, there is a higher affinity for pyrrolidinium-terminated dendrimers than for piperidinium-terminated dendrimers when there is a lower number of positive charges in the molecule: the affinity of AK72 (12 pyrrolidinium end groups) for siRNA was higher than for AK71 (12 piperidinium end groups), likely indicating a higher complexation stability of the pyrrolidinium-terminated dendrimer/siRNA dendriplexes than for the piperidinium-terminated dendriplexes. This higher stability indicates a higher complexation free energy, which is the sum of two contributions: enthalpic and entropic energies. This has been previously shown for pyrrolidinium end groups when compared to morpholinium groups.^37^ These differences in affinity are overcome when the number of positive terminal groups is increased to 24 for both pyrrolidinium and piperidinium. Interestingly, the presence of internal protonated amino groups (AK102) slightly reduced the potency of the phosphorus dendrimer to bind siRNA in spite of having 12 pyrrolidinium end groups.

To be used for *in vitro* and *in vivo* studies, siRNAs must be protected from RNase-mediated degradation.^38^ Thus, we tested the ability of these phosphorus dendrimers to protect siRNA and found that AK35 (5 μM) provided full protection against RNase-mediated degradation while AK71 (5 μM), AK72 (0.5 μM), and AK120 (0.5 μM), provided 50 to 80% protection. In turn, AK102 (5 μM) and AK116 (0.5 μM) only slightly protected siRNA against RNase-mediated degradation (Figure 2). However, these marked differences did not correlate well with the ability of these molecules to bind siRNA (Figure 1), since a lower number of terminal groups yielded a better protection of siRNA against RNase-mediated degradation, likely indicating a better coverage of the siRNA surface limiting the access of RNase to siRNA. This prompted us to hypothesize that the structural and physicochemical properties of the phosphorus dendrimers could correlate with their ability to protect siRNA from RNase-mediated degradation independent of their target affinity.^39^ We then decided to perform a molecular modeling study of AK35, AK71, and AK72 and their interactions with siRNA.

**Figure 2:**
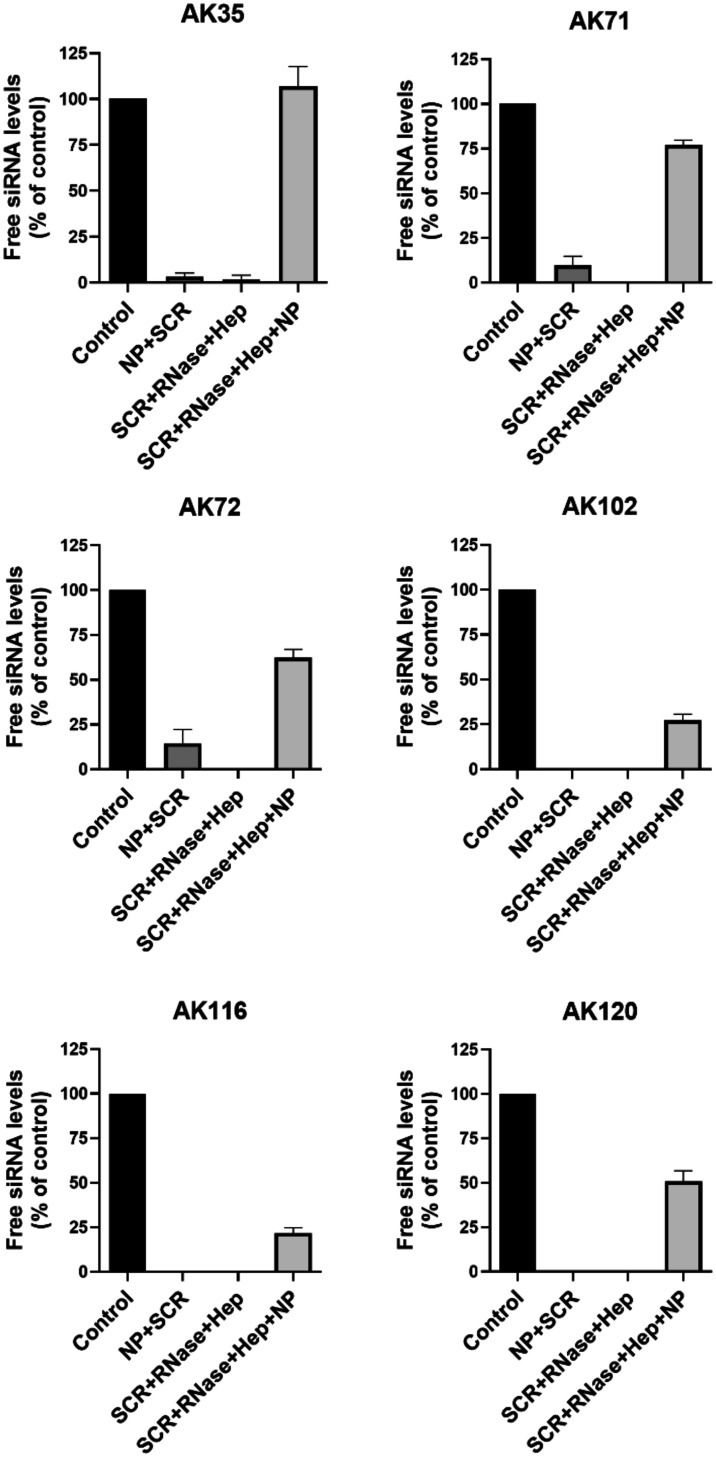
Phosphorus dendrimer-mediated protection from RNase-mediated degradation of siRNA. The experiment was performed as indicated in the Materials and Methods section. The following concentrations were used: AK35 (5 μm), AK71 (5 μm), AK72 (0.5 μm), AK102 (5 μm), AK116 (0.5 μm), and AK120 (0.5 μm). Data represent mean ± s.e.m of 3 independent experiments.

### Molecular Modeling

Conformational space, surface properties, and the MD of AK35 were studied by classical force field methods on three-dimensional all-atom models in three protonation states. AK35 includes six tertiary and six secondary amines in the branches. The tertiary amines were always modeled as protonated while the secondary amines were either all protonated, deprotonated, or three of the six homogeneously distributed around the N3P3 core were protonated. This study revealed that AK35 is a dendrimer of high conformational mobility and suggested that increasing protonation of AK35 modifies its shape and increases its size with radii of gyration (rGyr) from 7.5 over 8.0 to 8.6 Å as well as hydration of the branches and solvent access to the N3P3 core (Figures S1–S10). In addition, the conformational properties and interactions with solvent were similar in the MD simulations of models of AK71, AK72, and AK102 (Figure S13).

To study the interactions of phosphorus dendrimers with their target siRNA, the target was built as an all-atom model and subjected to MD simulations in explicit aqueous solution including 0.154 M KCl to challenge its stability and obtain conformations for molecular docking. Potassium (K^+^) and KCl were chosen for neutralization and provision of supplementary ions because K^+^ is the most abundant (earth-)­alkaline cation in the cytosol of mammalian cells and preferentially forms an ‘ionic atmosphere’ around RNA with transitory interactions rather than inducing specific RNA conformations or folds.^40^ On average, the siRNA model showed a volume (Vav) of 182,410 ± 589 Å^3^, rGyr of 19.9 ± 0.7 Å, and a longest end-to-end distance of 58 ± 4 Å (Figure S13). The central region of the double-stranded helix was stable in terms of base pairing and helical parameters while losses of canonical pairing were observed at the 5′ and 3′ ends. The higher conformational flexibility at the 5′ and 3′ ends compared to the central regions of double-stranded RNA (dsRNA) we observed is typical in MD simulations.^41^

Docking of AK35, AK71, and AK72 to the central regions of our target siRNA revealed the complete spectrum of possible interactions of these dendrimers and this siRNA. All three phosphorus dendrimers bound to the inner major groove, meaning that this binding could be specific. Although AK72 showed greater affinity in the gel retardation assay compared to AK35 and AK71, of the three, it provided the least protection against RNase-mediated degradation. AK35 provided full protection against degradation but did not group with the strongest binders. In the docking study, AK35 stood out because it capped the 5′ and 3′ ends of the siRNA, as opposed to AK71 and AK72 which did not present similar capping but docked in the inner major groove at the 5′ and 3′ ends, as observed in the central regions (Figure S12). Given that RNases degrade siRNA starting from the ends, the difference in capping as revealed by molecular docking was striking.

We chose four representative models issued from the MD simulation to explore the structural variability at the 5′ and 3′ ends in terms of interactions with phosphorus dendrimers (for more details, see Supporting Information). AK35 docked to the central regions of the target siRNA always in the major groove, regardless of which of the three protonation states was considered. In these poses the N3P3 core did not directly interact with siRNA, while the branches formed interactions with both strands A and B (Figure 3). Thus, the dendrimer bridged the phosphate backbones of the RNA strands over the major groove by electrostatic interactions of protonated secondary and tertiary amines. The branches also hydrogen bonded through neutral amines in addition to forming π–π-stacking, π-cation interactions, and classical and aromatic hydrogen bonds to RNA bases (Figure 3). Hence, the dendrimer smoothly adhered to the inner face of the helical RNA ribbon by forming interactions with all six branches.

**Figure 3:**
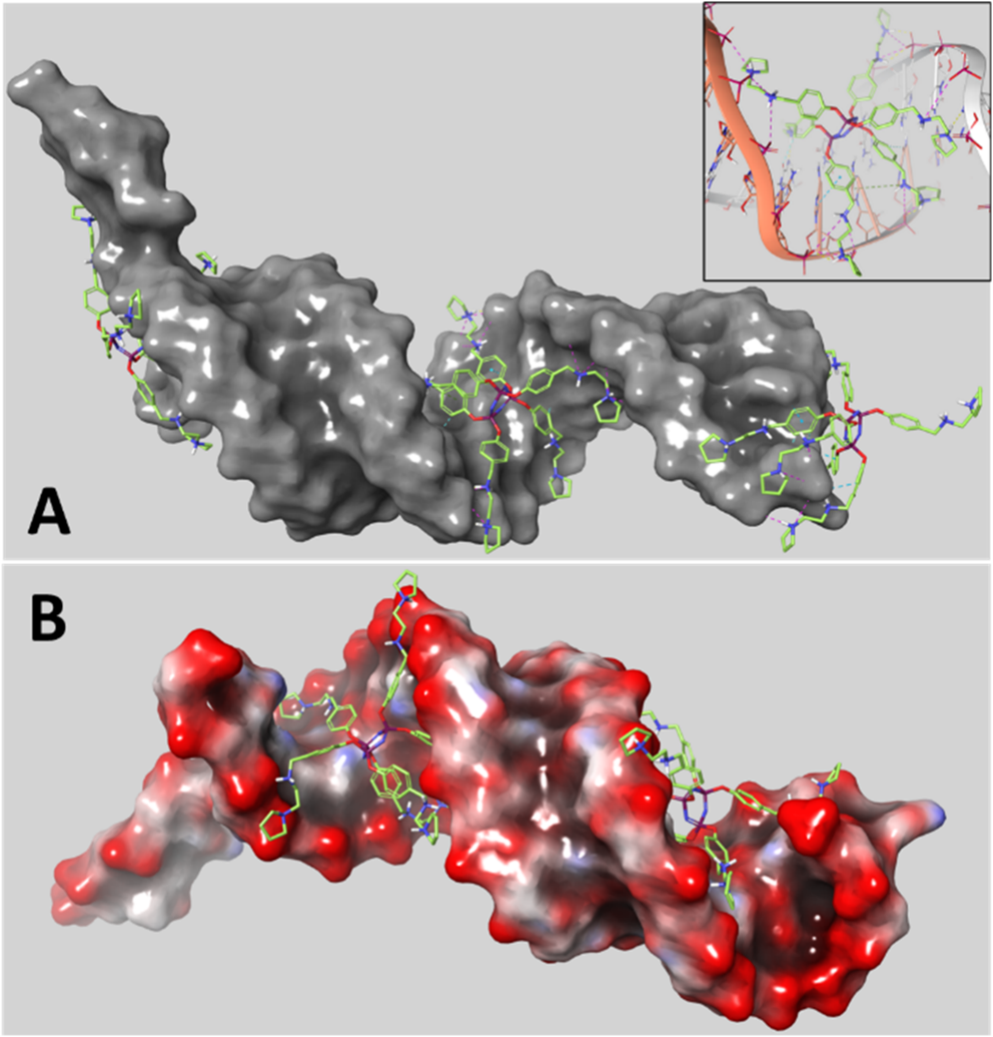
Three-dimensional models of fully protonated AK35 docked to the center, 3′- and 5′-ends of conformation 1 of siRNA obtained by a MD simulation. The positively charged secondary and ternary amine groups of the branches formed numerous ion pair interactions with the negatively charged phosphate groups of RNA strands A and B. Nevertheless, π–π-stacking, π-cation interactions, classical and aromatic hydrogen bonds between dendrimer branches and RNA bases were also observed; inset (A): close-up of the center docked pose. siRNA was represented by its SASA colored in gray (A) or by electrostatic potential from negative over neutral to positive in red over white to blue (B). The chemical structures of dendrimer and siRNA ((A) inset) were depicted as tubes with oxygen, nitrogen, phosphor, and hydrogen atoms indicated in red, blue, purple, and white, respectively; carbon atoms were depicted in green and white or brown for AK35 and RNA, respectively; nonpolar hydrogen atoms were not represented for clarity; backbone trace and base orientation of siRNA were visualized by ribbons and sticks. Noncovalent interactions are indicated by dashed lines: salt bridges—magenta; classic and aromatic hydrogen bonds—yellow and light blue, rsp; perpendicular or parallel π-π-stacking—cyan; π-cation—green.

Docking of the AK35 models to the 5′ and 3′ ends of the four conformational siRNA representatives revealed a similar picture: all hydrogen bond, electrostatic, and π-interaction types were observed when the dendrimer embraced the ends of the helical ribbon to interact with phosphate, ribose, and nucleobase units (Figures 3 and S11). Interactions were more often formed on the inner major groove face of the RNA ribbon than on the outer minor groove face. While not all AK35 branches interacted simultaneously with the target in all of these poses, direct hydrogen bonding of the N3P3 core to a terminal adenine occurred (Figure S11A).

Docking of AK71 and AK72 was also performed: while they bound to the central region of the siRNA in the same manner as AK35, surprisingly few poses were obtained when docking to the 5′ and 3′ ends of the target. Moreover, these poses differed from those of AK35: instead of capping the siRNA ends, AK71, and AK72 were entirely placed in the major groove and interacted, as observed, for the poses docked to the central regions of the siRNA (Figure S12). To explore phosphorus dendrimer interactions with their target siRNA further, we constructed a AK35/siRNA dendriplex of stoichiometry 13:1 and challenged it by two MD simulations for 1 μs in aqueous solution including 154 mM KCl at 300 K and 1 atm. Early in both calculations, three AK35 molecules diffused away, the resulting dendriplex maintained 10:1 stoichiometry for more than 900 ns until the end of the simulations. The solvent accessible surface area (SASA) of the dendriplex arose from not only AK35 but also siRNA. AK35 resided predominantly in the major inner groove and only partially covered surface areas of the minor outer groove. Hence, the AK35/siRNA dendriplex did not only present a charge distribution fluctuating between neutral and positive electrostatics arising from AK35 SASA, but large zones of neutral to negatively charged SASA were contributed by the minor groove (Figure S14). For further details, see Supporting Information.

All the above suggested that the intrinsic higher flexibility expressed by the higher number of rotors and lower cyclization degree of the AK35 branches compared to AK71 and AK72 (Table S1) was likely responsible for better protection against RNase-mediated degradation. Therefore, these observations suggested that the intrinsic flexibility of dendrimer branches was an independent factor that influenced affinity and protection against RNase-mediated degradation that should be considered alongside the chemical structure. More generally, these data revealed that affinity did not provide protection per se but rather, the mode of binding to specific regions of siRNA played an important role in enzymatic degradation.

The interactions between small molecules and proteins with dsRNA have been studied both through theoretical approaches and experimentally. MD simulations of dsRNA in the presence of hexamethylenetetramine revealed preferential binding of the cation in the major groove in contrast to double-stranded DNA–RNA hybrids (dsDRNA) or DNA (dsDNA) which preferentially bound the cation at the phosphate backbone edge, outside of the major groove.^42^ Indeed, the stronger negative electrostatic potential of the major groove of dsRNA compared to dsDRNA, was recognized as the driving force of the different binding preferences in concert with the specific helical parameters of the three nucleic acid types.

Aminoglycoside antibiotics represent the paradigm for the differentiation of the structure–activity relationships of RNA-binding small molecules in terms of biological mechanisms of action, affinity, and selectivity for prokaryotic, protozoal, or eukaryotic rRNA A-sites. They bind in the inner major groove, predominantly interacting with the nucleobases. One of them, NB33, stands out because its exceptional conformational flexibility was recognized as the driving force for its unusual biological activity profile.^43^

Thus, the case of NB33 reinforces our interpretation that AK35, although not the strongest binder, better protects siRNA from RNase-mediated degradation because of its specific conformational freedom which caps the siRNA ends. Aminoglycosides show that binding mode, affinity, and biological activity are independently ruled by structure and the physicochemical properties of RNA-interacting agents. Phosphorus dendrimers show similar subtle differences in various biological assays, in agreement with the aminoglycoside paradigm. For a detailed discussion of this, along with references, please see Supporting Information.

While these small molecules locate and preferentially interact with both the nucleobases and phosphates of the RNA backbone in the major groove of dsRNA, the interaction schemes of dsRNA with proteins such as, for example, p19 of tombusvirus or transactivation response RNA binding protein (TRBP2) differ. These proteins wrap around the outer face of the dsRNA ribbon to predominantly interact with the phosphate backbone and ribose units, either directly or mediated by water molecules. Furthermore, at the 5′ and 3′ ends, whether blunt or with overhangs, they may form direct interactions with the nucleobases.^19,44^ argonaute (AaAgo) binds to the two-nucleotide overhang of 22-mer and 26-mer siRNA at one 3′ end by specific interactions which are proposed to be of biological relevance. At the other end of the siRNA only nonspecific packing—presumably without biological relevance—is observed.^20^ In addition, crystal structures of Argonaute (TtAgo), in ternary complexes with guide-DNA and target-RNA, form a dsDRNA hybrid in A-form, with the protein, again, wrapping around the outer face of the helix and interacting with the phosphate-ribose backbone or nucleobases from the side of the minor groove.^45^

The phosphorus dendrimers considered in our study have molecular weights between 1400 and 4200 g/mol which are thus, more than twice that of aminoglycoside antibiotics whose molecular weight ranges between 440 and 720 g/mol, or hexamethylenetetramine at 140 g/mol, but are still far below the molecular weight of small proteins such as p19 (14 kDa). They generally feature incomparable conformational flexibility due to their dendrimer architecture when compared to both the linear or branched sugar chains of aminoglycosides and properly folded proteins, not to mention hexamethylenetetramine. This is especially the case for AK35 and, by extension, also for AK102. Compared to aminoglycoside antibiotics and hexamethylenetetramine, their chemical structures also present a larger spectrum of interaction types including not only classic hydrogen bonding or ion pairs, but also aromatic interaction potential.

Furthermore, the chemical structures of AK35 and AK102, which comprise secondary and tertiary amines, provide extraordinary combinatorial and spatial diversity of positive charge repartitioning which allows them to adapt to their target in function of pH. Our docking study suggests that binding to the central region of double-stranded siRNA will target the major groove, just like aminoglycoside antibiotics and hexamethylenetetramine, thereby having the potential of selectivity and preferential binding to sequence specific regions of siRNA. Nonetheless, binding to the 5′ and 3′ ends of target siRNA can also occur by aromatic, electrostatic, and hydrogen bond interactions that may even implicate the central N3P3 core, as extensively described for proteins interacting with dsRNA or dsDRNA hybrids. Thus, phosphorus dendrimers unite the siRNA-binding capacities of both small molecules and proteins in a specific and unprecedented manner and thereby open an entire field to RNA-implicating drug discovery and delivery.

### Biophysical Characterization

As shown in Table 1, all siRNA-dendrimer complexes exhibit a decrease in ζ-potential compared to their respective pristine dendrimers (except for AK116). Despite this decrease, ζ-potentials remain positive, indicating that the complexation of the siRNA with the dendrimers does not fully neutralize the dendrimer surface charges, a beneficial feature for maintaining stability and promoting cell interaction. Notably, the ζ-potential of AK116 increases from 33.9 to 40 mV, suggesting either a stronger electrostatic interaction with siRNA or a possible surface restructuring upon complexation.

**Table 1:** Zeta Potential (ζ-Potential), Average Hydrodynamic Diameter, and Polydispersity Index (PdI) for Dendrimers and Their Complexes with siRNA

sample	ζ-potential (mV)	average hydrodynamic diameter (nm)	polydispersity index (PdI)
AK35	35.2 ± 1.5	320.4 ± 30.3	0.351
AK71	33.0 ± 2.6	183.3 ± 10.1	0.295
AK72	24.2 ± 1.1	244.1 ± 30.0	0.273
AK102	18.1 ± 1.8	227.8 ± 1.7	0.270
AK116	33.9 ± 2.3	293.2 ± 15.5	0.310
AK120	34.4 ± 3.1	410.6 ± 46.4	0.414
siRNA:AK35	30.6 ± 0.8	158.2 ± 0.4	0.062
siRNA:AK71	29.4 ± 0.6	187.4 ± 5.1	0.231
siRNA:AK72	30.6 ± 0.5	150.1 ± 4.3	0.106
siRNA:AK102	26.2 ± 1.5	222.4 ± 35.7	0.322
siRNA:AK116	40.0 ± 1.9	109.9 ± 6.8	0.261
siRNA:AK120	34.6 ± 1.1	129.3 ± 7.5	0.319

In most cases, complexation with siRNA led to reduce the size of the particles, which may indicate a decrease in aggregation. The polydispersity index (PdI) also decreased significantly, especially for AK35, which dropped from 0.351 to 0.062, indicating highly uniform particles after binding with siRNA. For AK102, complexation with siRNA did not change the size of particles, while for AK116 and AK120 the size substantially dropped by 183 and 291 nm, respectively. This indicates much stronger interaction with siRNA in the latter two dendrimers as expected by the presence of a higher number of positively charged groups (24) in these two dendrimers.

To investigate the chiroptical properties and secondary structure of the dendrimers and their complexes, we employed circular dichroism (CD) spectroscopy. As evidenced in Figure S15a, none of the dendrimers exhibited any CD signal, which was expected due to the absence of chiral centers in these molecules. However, their complexes with siRNA displayed a strong ellipticity around 267–270 nm, characteristic of a helical secondary structure of siRNA (Figure S15b). The presence of this CD signal confirmed successful siRNA complexation. Variations in the intensity of CD signal might indicate differences in siRNA loading or binding efficiency. Also, a slight blue shift was observed: the signal shifted from 271 nm for AK35, to 270 nm for AK72, to 268 nm for AK71 and AK102, to 267 nm for AK116, and to 266 nm for AK120.

### Toxicity

The intrinsic toxicity of phosphorus dendrimers was evaluated in several tumor cell lines, including two glioblastoma (GBM) lines, as well as in primary astrocyte cultures—a cell type that shares anatomical space in the brain with GBM cells. Cells were exposed to increasing concentrations of dendrimers ranging from 0.1 to 10 μM (Figure 4). In T98G cells, neither AK35 nor AK102 exhibited toxicity up to 10 μM. In contrast, AK71 and AK72 showed marked toxicity at 5 and 10 μM, with a sharp increase between 1 and 5 μM. AK116 also demonstrated significant toxicity at 10 μM, with a steep rise observed between 5 and 10 μM. For AK120, only a slight increase in toxicity was detected at 10 μM. A comparable toxicity profile was observed in astrocytes: AK35 and AK102 remained nontoxic up to 10 μM, while AK71 induced toxicity similar to that observed in T98G GBM cells. However, the remaining four phosphorus dendrimers produced higher levels of toxicity in astrocytes (Figure S16). Additionally, IC_50_ values for inhibition of cell proliferation were determined for the various dendrimers across a panel of commercially available tumor and nontumor cell lines (Table 2). These data were consistent with observations in T98G cells, confirming that AK35 and AK102 were nontoxic to the U87-MG GBM cell line. In general, the other dendrimers were more toxic to tumor cell lines than to nontumor cells. Notably, AK72 deviated from this pattern, exhibiting greater toxicity in nontumor cells (Table 2).

**Figure 4:**
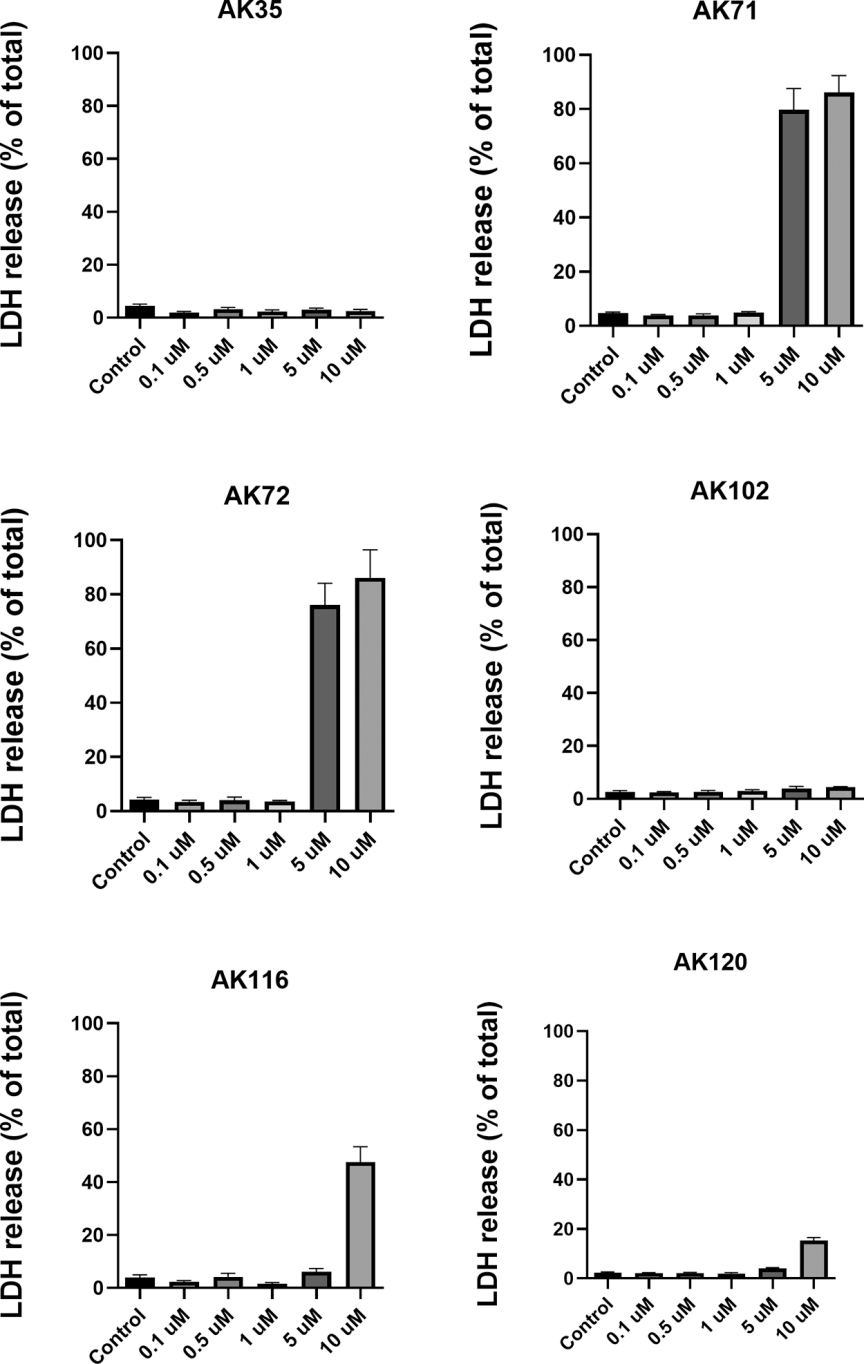
Toxicity of cationic phosphorus dendrimers on T98G human glioblastoma cells. Cells were exposed to the indicated dendrimer concentrations for 72 h and toxicity was measured as LDH release to the culture medium as indicated in the Materials and Methods section. Data represent mean ± s.e.m of 8 to 12 experiments.

**Table 2:** Activity of the Cationic Phosphorus Dendrimers against a Panel of Tumoral and Nontumoral Cell Lines^a^

	IC_50_ (μM)							
	HCT116	U87-MG	U2OS	A549	K562	MDA-MB231	MRC5	RPE1
AK35	37 ± 3.30	nd	nd	130 ± 57	39.8 ± 4.2	nd	nd	nd
AK71	1 ± 0.01	8.73 ± 0.54	0.26 ± 0.04	6.6 ± 0.16	0.86 ± 0.0007	0.68 ± 0.01	9.9 ± 0.01	9.82 ± 0.30
AK72	6.47 ± 0.28	1.09 ± 0.05	2.68 ± 0.16	6.3 ± 0.32	2.98 ± 0.17	1.18 ± 0.06	1.06 ± 0.03	0.61 ± 0.05
AK102	10 ± 0.18	nd	9.7 ± 0.34	10.5 ± 0.21	20.7 ± 0.79	10.37 ± 0.85	51.5 ± 6.84	49.9 ± 1.00
AK116	8.7 ± 0.3	2.56 ± 0.58	8 ± 0.78	8.4 ± 0.71	8.24 ± 0.74	3.22 ± 0.30	5.42 ± 0.05	5.83 ± 0.20
AK120	1.88 ± 0.22	17.2 ± 0.09	9.97 ± 0.04	4.85 ± 0.64	9.56 ± 0.27	1.47 ± 0.03	6.41 ± 0.26	11.9 ± 0.57

a Tumoral cells: HCT-116 colorectal carcinoma, U2OS osteosarcoma, MDA-MB231 breast carcinoma, A549 lung carcinoma, K562 leukemia, U87-MG glioblastoma. Non-tumoral cell: MRC-5 derived from normal lung, RPE1 immortalized retinal pigment epithelial cells. Data represent men ± s.e.m of 6 to 12 experiments.

We observed that the phosphorus dendrimers AK71 and AK72, which bear 12 piperidinium or pyrrolidinium groups, respectively, exhibited higher toxicity against tumor cell lines compared to other phosphorus dendrimers. Of note, it is generally accepted that the more positive charges a nanoparticle has, the higher its toxicity.^46^ However, this does not appear to be the case for cationic phosphorus dendrimers. For example, AK120 and AK116, which bear 24 terminal piperidinium or pyrrolidinium groups, respectively, were less toxic than AK71 and AK72 (which have only 12 of these groups), suggesting that factors beyond the total number of positive charges contribute to dendrimer toxicity.

In contrast to AK102 and AK35, increased toxicity was observed in primary astrocytes—the healthy, nontumoral counterpart of GBM cells—for AK72, AK102, AK116, and AK120. Notably, AK102, which contains 12 pyrrolidinium end groups like AK72, did not exhibit toxicity toward either GBM cell lines or astrocytes (Figure S16), further indicating that the number of positive charges alone does not determine toxicity. Another important factor for intrinsic nanoparticle toxicity is the cell type where the presence of mechanism of uptake/transport for the nanoparticle to enter the cell can contribute to toxicity facilitating nanoparticle entry and accumulation. Another factor related to the previous one is the nanoparticle aggregation that increases the size and reduces uptake. As it can be observed in Figure 4 and Table 1, in general, the nanoparticles with a larger hydrodynamic radius: AK35 and AK120 are less toxic that dendrimers with a smaller hydrodynamic radius

Although AK102 has 24 secondary or tertiary amines that can be protonated, the spatial arrangement of these protonatable groups within a single branch is very similar to that of AK35. At physiological pH, only about half of the amines are protonated, which prevents protonation of the second amine on the same branch. The situation differs for AK71, AK72, AK116, and AK120, where the protonatable nitrogen atoms are spaced farther apart; these dendrimers are more likely to be fully protonated at physiological pH. In fact, experiments conducted with AK35—the only phosphorus dendrimer with six linear branches connected to the P_3_N_3_ core, in contrast to the double-branched terminal units of all the others—clearly highlight the importance of internal structure. AK35 exhibited no toxicity to either GBM cells or primary astrocytes. These data also suggest that piperidinium end groups are less toxic than pyrrolidinium end groups.

After testing all selected compounds across a panel of cancer cell lines, we found that the most active dendrimers had IC_50_ values in the submicromolar range (Table 2). Interestingly, there was little difference in cytotoxic activity between AK71 (12 piperidinium end groups) and AK72 (12 pyrrolidinium end groups) across five selected tumor cell lines. In T98G cells, toxicity was observed at concentrations higher than those required for effective siRNA binding and protection, making these dendrimers promising candidates for transfection studies.

### Hemolysis

To evaluate the potential biocompatibility of the phosphodendrimers, we investigated their possible hemolytic effects. To this end, mouse RBCs were isolated and exposed to various concentrations of either dendrimers alone or dendriplexes. As shown in Figure S17, only AK102 exhibited slight toxicity at the concentrations used for transfection: AK35 (5 μM), AK71 (1 μM), AK72 (0.5 μM), AK102 (5 μM), AK116 (0.5 μM), and AK120 (0.5 μM). This indicates generally good biocompatibility of the phosphodendrimers. We further explored the hemolytic effect of dendriplexes formed by the phosphorus dendrimers at concentrations higher than those used in transfection studies: AK35 (10 μM), AK71 (1 μM), AK72 (10 μM), AK102 (1 μM), AK116 (1 μM), and AK120 (1 μM), combined with three different concentrations of scrambled siRNA (10, 25, and 50 nM). As shown in Figure S18, no hemolysis was observed, further supporting the biocompatibility of the dendrimers.

### SIRNA Delivery to Glioblastoma Cells

The ability of cationic phosphorus dendrimers to deliver siRNA into cells and facilitate their endosomal escape was studied using fluorescent siRNA. As shown in Figure 5, after 6 h of treatment with dendriplexes comprising different phosphorus dendrimers (AK35, 5 μM; AK71, 1 μM; AK72, 0.5 μM; AK102, 5 μM; AK116, 0.5 μM; and AK120, 0.5 μM) and FAM-labeled siRNA (100 nM), only a very weak signal was observed in the T98G cells in the case of AK71 and AK120, with no signal being detected for the other dendrimers. The signal in the T98G cells was significantly lower than that provided by the β-cyclodextrin derivative, AMC11 ^6^ used as a siRNA delivery control.

**Figure 5:**
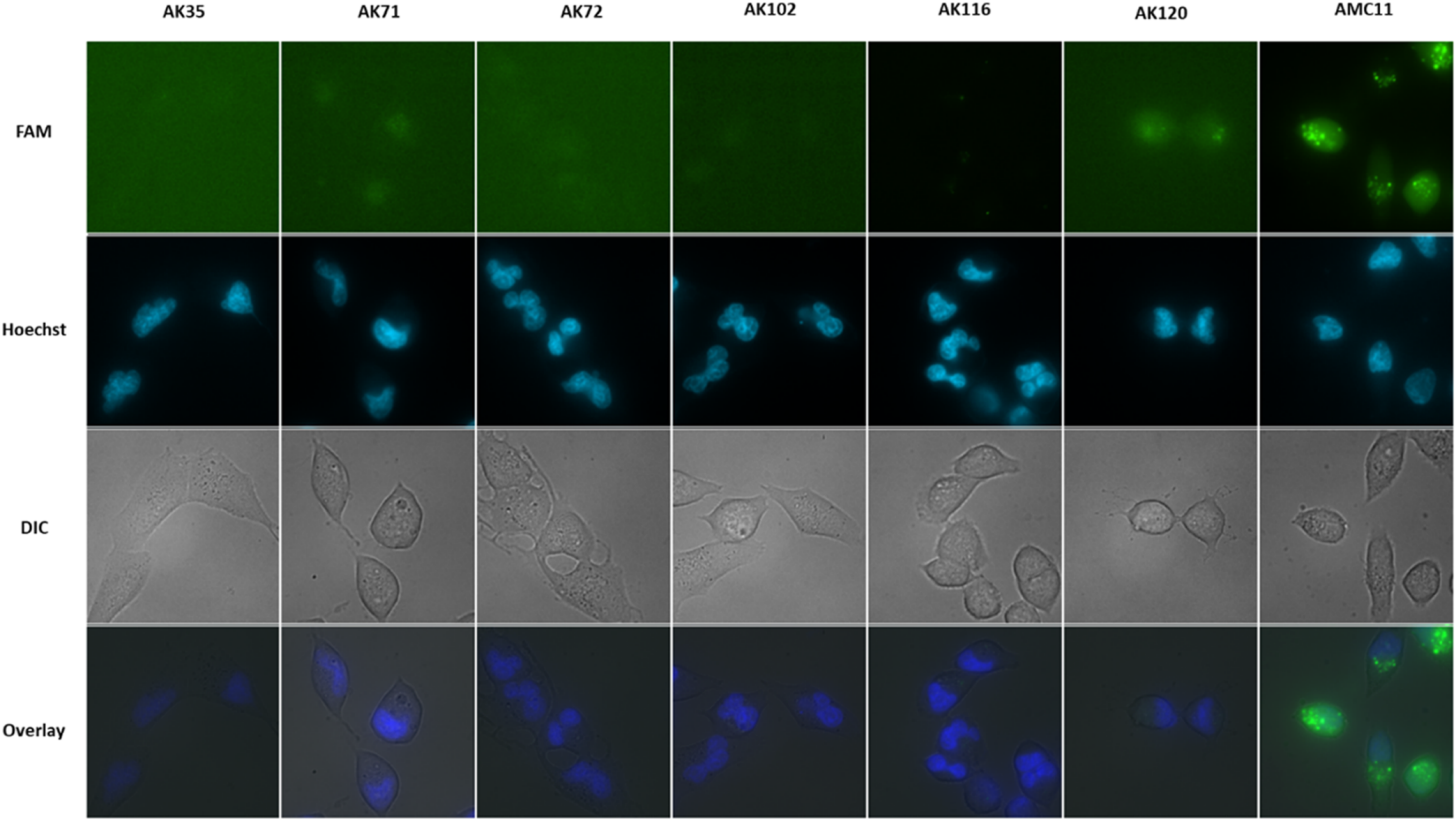
siRNA delivery to T98G glioblastoma cells by phosphorus dendrimers. T98G glioblastoma cells were incubated for 6 h with dendriplexes containing either cationic phosphorus dendrimers: AK35 (5 μM), AK71 (1 μM), AK72 (0.5 μM), AK102 (5 μM), AK116 (0.5 μM), and AK120 (0.5 μM) or the β-cyclodextrin derivative AMC11 plus FAM-siRNA (100 nM) for 8 h. Ten minutes before recording the data, Hoechst 33342 (25 μg/mL) was added to the culture medium. The figure shows representative images of FAM-siRNA, Hoechst 33343 staining the nuclei, overlay of FAM-siRNA, and differential interference contrast microscopy (DIC) image.

However, neither AK71 nor AK72 was able to transport fluorescent siRNA into GBM T98G cells and, accordingly, could not significantly decrease the p42-MAPK (Figure 6).

**Figure 6:**
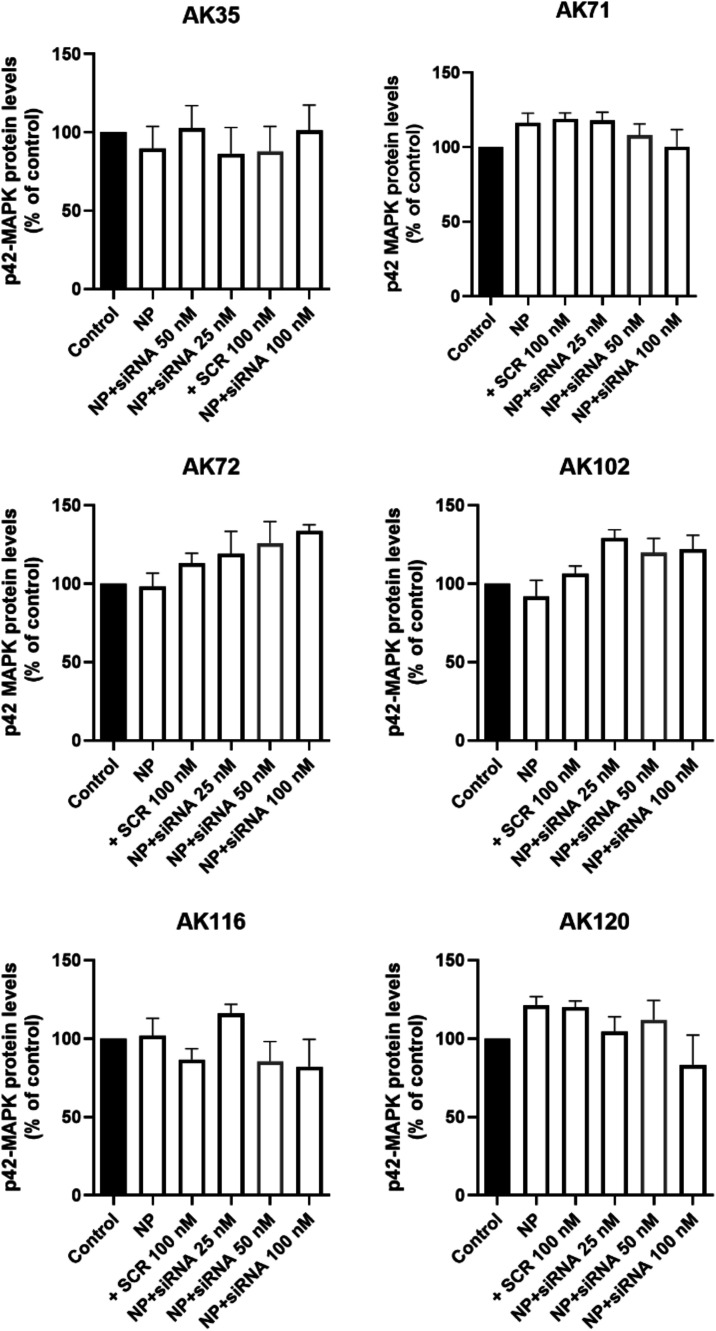
Transfection efficiency of specific siRNA by different phosphorus dendrimers to knock down p42-MAPK in T98G human glioblastoma cells. T98G glioblastoma cells were incubated for 72 h with dendriplexes containing the indicated cationic phosphorus dendrimers: AK35 (5 μM), AK71 (1 μM), AK72 (0.5 μM), AK102 (5 μM), AK116 (0.5 μM), and AK120 (0.5 μM) plus specific siRNA (100 nM) against p42-MAPK or noncoding scramble siRNA (SCR). Cellular protein levels of p42-MAPK were detected by Western blot as indicated in Methods. Data represent mean ± s.e.m. of 5 experiments. Control represents the protein levels in untreated cells taken as reference value (100%).

There may be several causes for this, with the lack of dendriplex uptake by the cells being the most likely. Several physical properties can influence nanoparticle uptake by cells, including size, with uptake being higher for smaller nanoparticles.^47^ The hydrodynamic radius of 1-G1 cationic phosphorus dendrimers in complex with their target plasmid DNA (pDNA)-encoding enhanced green fluorescent protein (EGFP) measuring about 240 amino acids, or the tumor suppressor p53, measuring about 400 amino acids, is 200 to 300 nm,^48^ which is close to the estimated upper limit of 200 nm for endocytotic uptake,^29^ However, it is still below the upper size limit of 1 μm for macropinocytosis,^49^ a mechanism used by NPs to get access to the cell interior which has been described in GBM cell lines from different animal species.^29^ Nevertheless, both EGFP and p53 were successfully intracellularly delivered as dendriplexes with 1-G1 when prepared at a molar ratio of 20 positive charges per phosphate group.^48^

Our target siRNA has a longest *d*_end‑end_ of 5.8 nm while the phosphorus dendrimers show longest *d*_end‑end_ of 1.4 to 1.9 nm in MD simulations (see Supporting Information). Assuming monodisperse solution of one siRNA complexed with one layer of phosphorus dendrimer suggests 7.7 nm as upper limit of the longest *d*_end‑end_ with a hydrodynamic size in the same order of magnitude. Thus, siRNA dendriplexes are much smaller than pDNA dendriplexes and their cellular uptake should be expected. However, phosphorus dendrimers condense pDNA in dendriplexes^48^ while much smaller siRNA of only 42 nucleobases does not. Thus, dendriplex density and stability should be higher in pDNA complexes with phosphorus dendrimers compared to siRNA complexes.

Although there is no general agreement on the role of nanoparticle shape in the uptake process, it seems that nanoparticle rigidity contributes to facilitating cellular uptake.^37^ Furthermore, cationic nanoparticles are better internalized than neutral or negatively charged nanoparticles.^50^ Our MD simulations of AK35/siRNA dendriplex suggest that ten AK35 molecules each modeled with nine positive charges may stably bind to one siRNA under physiological conditions. This stoichiometry generates an overhang of 71 positive charges in the dendriplex and cellular uptake may be expected. However, these positive charges are not uniformly distributed on the SASA of the dendriplex. Its positively charged SASA is fragmented by large patches of negatively charged SASA provided by the minor groove. Thus, on the one hand, siRNA–phosphorus dendriplexes might not present sufficient uniformly distributed positive charge density, stability, and rigidity compared to pDNA dendriplexes. However, on the other hand, polymer flexibility plays a relevant role in siRNA binding.^51^ Thus, a balance between flexibility to bind siRNA and a certain level of rigidity to facilitate uptake would be required for effective siRNA transfection efficiency. Finally, the interaction with serum proteins triggering the formation of a protein corona over the NP surface could also contribute to decreasing the cellular uptake of the dendriplexes.^52^

### Transfection Efficiency of Phosphorus Dendrimers

We tested the different phosphorus dendrimers for their transfection efficiency using two specific siRNAs designed against p42-MAPK, a protein involved in the proliferation and survival of GBM cells. As shown in Figure 6, none of the cationic phosphorus dendrimers/siRNA dendriplexes decreased the p42-MAPK protein levels, thereby indicating a lack of transfection efficiency. This was in sharp contrast with the high transfection efficiency of AMC11, which decreased p42-MAPK protein levels in GBM cells to about 20% of the control values (Figure S19) as previously described for the human prostate cancer cell lines PC3 and LnCaP.^6^

## Conclusions

In summary, this new family of cationic phosphorus dendrimers presents good siRNA binding with those bearing terminal pyrrolidinium having the highest affinity and, in general, significantly protects siRNA from RNase-mediated degradation. Molecular modeling revealed high levels of conformational flexibility that allowed the compounds to adjust their shapes to the target and smoothly adhere to the inner major groove of siRNA by deploying the full spectrum of noncovalent interactions permitted by their chemical structures. It also indicated that these dendrimers conform to the paradigm of aminoglycosides interacting with dRNA. Thus, intrinsic flexibility and individual binding properties contribute to the modulation of the biological profile beyond overall protonation status. They are toxic to GBM T98G cells but at concentrations above those required for siRNA binding. However, none of the phosphorus dendrimers was able to deliver siRNA into GBM cells and, accordingly, did not show significant transfection efficiency. These results suggest that although these cationic dendrimers have interesting properties for siRNA transfection, modifications of these molecules will be required to efficiently deliver siRNA into the target cells. These modifications might include the incorporation of aliphatic chains of different lengths to increase amphiphilicity to facilitate crossing of the cell membrane. In addition, decoration of the dendrimers with cell-permeating peptides can contribute to dendrimer entry and siRNA transport into the cells.

## Supplementary Material



## Notes

Procedure to prepare the polycationic phosphorus dendrimers; structure and NMR data for compounds; detailed material and methods for molecular modeling; additional results and discussion of molecular modeling (Figures S1–S14 and Table S1); circular dichroism (Figure S15); hemolysis studies (Figures S16–S17); toxicity of cationic phosphorus dendrimers on primary mouse astrocytes (Figure S18); transfection efficiency of AMC11 on p42-MAPK protein levels (Figure S19) (PDF)

## Abbreviations

AaAgo: argonaute
BCA: bicinchoninic acid
BSA: phosphate-buffered saline
DIC: differential interference contrast microscopy
Dulbecco’s: minimal essential médium
dsRNA: double-stranded RNA
dsDNA: double-stranded DNA
dsDRNA: double-stranded DNA–RNA hybrids
ECL: enhanced chemiluminescence
EDTA: ethylenediamine tetra-acetic acid
EGFP: enhanced green fluorescent protein
FDA: Food and Drug Administration
FCS: fetal calf serum
GBM: glioblastoma
GAPDH: glyceraldehyde 3-phosphate dehydrogenase
HEPES: *N*-2-hydroxyethylpiperazine-*N*′-2-ethanesulfonic acid
KH: Krebs–Henseleit solution
LDH: lactate dehydrohgenase
MD: molecular dynamics
p42-MAPK: p42-mitogen-activated protein kinase
PAMAM: polyamidoamine
PBS: phosphate-buffered saline
PDB: protein data bank
rGyr: radii of gyration
RNAi: interference RNA
RNase: ribonuclease A
siRNA: small interference RNA
SASA: solvent accessible surface área
SDS-PAGE: sodium dodecyl sulfate polyacrylamide gel electrophoresis
SPC: simple point charge
TMZ: Temozolomide
TRBP2: transactivation response RNA binding protein
TtAgo: argonaute
