# Supplementary material for: siRNA Interaction and Transfection Properties of Polycationic Phosphorus Dendrimers
Source: Biomacromolecules. 2025 May 30;26(7):4158–73. doi: 10.1021/acs.biomac.5c00171 (PMC12264960; doi:10.1021/acs.biomac.5c00171)
Supplement: Supplementary file 1 [file bm5c00171_si_001.pdf]

siRNA interaction and transfection properties of polycationic phosphorus dendrimers

Irene Rodríguez-Clemente[1,2], *Andrii Karpus*[3], *Angel Buendía*[1,2], *Krzystof Sztandera*[1,2], *Elzbieta Regulska*[3,4], *Jerome Bignon*[5], *Anne-Marie Caminade*[5], *Carlos Romero-Nieto*[3,4], *Anke Steinmetz*[6*], *Serge Mignani*[7,8*], *Jean-Pierre Majoral*[5*], *and Valentín Ceña*[1,2*#]

[1]Unidad Asociada Neurodeath. INAMOL. Universidad de Castilla-La Mancha, Albacete, 02006 Spain; [2]CIBER, Instituto de Salud Carlos III, Madrid, 28028, Spain; [3]Department of Inorganic, Organic and Biochemistry, University of Castilla-La Mancha, Albacete, 02006, Spain ; [4]IRICA. Universidad de Castilla-La Mancha, Ciudad Real, 13071, Spain; [5]Laboratoire de Chimie de Coordination, CNRS, Toulouse, 31077 cedex 4, France; [6]Sanofi R&D, CMC Synthetics/BTDV/EB, CRV, Vitry-sur-Seine, 94400, France ; [7]Centre d'Etudes et de Recherche sur le Medicament de Normandie (CERMN), Université de Caen Normandie, Caen 14032, France; [8]CQM - Centro de Química da Madeira, MMRG, Universidade da Madeira, Funchal, 9000·390**,** Portugal.

*Co-corresponding authors

[#]To whom all correspondence should be addressed

Index

1. Typical procedure to prepare the polycationic phosphorus dendrimers 3

2. Structure and NMR data for compounds.. 4

3. Molecular Modeling...8
   Materials & Methods 8
   Results 9
   Discussion 14

4. Circular dichroism..27

5. Toxicity of cationic phosphorus dendrimers on primary mouse astrocytes..................28

6. Hemolysis studies of dendrimers and dendriplexes 29

7. Transfection efficiency of AMC11 on p42-MAPK protein levels...............................31

8. References… 32

1. Typical procedure to prepare the polycationic phosphorus dendrimers

A combined mixture of chlorinated phosphorus dendrimer prepared according to scheme 1, pyrrolidine or piperidine and potassium carbonate in acetonitrile was stirred at 90°C for 20hrs. After elimination of potassium carbonate by filtration through celite, the resulting solution was concentrated in vacuo leading to a yellow oil which was washed several times with 50ml of methanol to give the neutral phosphorus dendrimers AK71 orAK72 which were dried under high vacuum AK71 (12 terminal piperidinium groups) or AK 72 (12 terminal pyrolidinium groups) were obtained respectively in 85% and 82% yield. Addition of HCl (1M) to a THF solution of these neutral phosphorus dendrimers at 0°C followed by stirring the resulting solution at RT for 1hr afforded the polycationic phosphorus dendrimers AK 71 or AK 72as white powders in near quantitative yields.

1 The same processes were applied for the synthesis of the neutral then cationic phosphorus dendrimers AK116 (24 terminal pyrrolidinium groups) or AK 120 (24 terminal piperidinium groups) in 75% yield for the neutral form and the corresponding cationic forms.

2. Structure and NMR data for compounds AK35, AK71, AK72, AK102, AK116, AK120

Compound AK 35

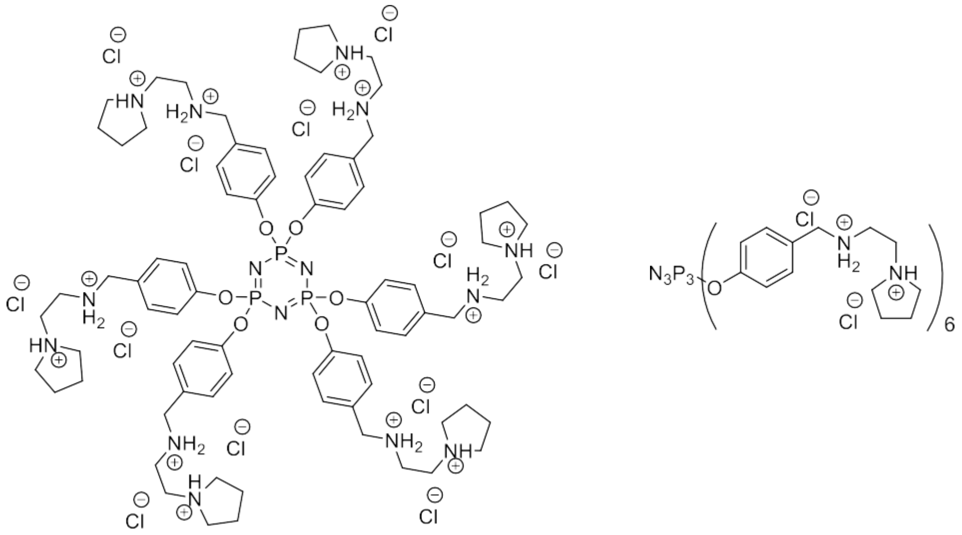

$C_{78}H_{126}Cl_{12}N_{15}O_6P_3$, M_r = 1888.29 g/mol. ^{1}H NMR (400MHz, D_2O) δ (ppm): 7.42 (12H, d, $^3J_{HH}$ = 6.0 Hz, ^{Ar}C*H*), 6.98 (12H, d, $^3J_{HH}$ = 8.3 Hz, ^{Ar}C*H*), 4.31 (12H, s, ^{Ar}C-C*H$_2$*-NH), 3.62-3.54 (12H, m, NH-C*H$_2$*-CH$_2$-N), 3.54-3.45 (12H, m, NH-CH$_2$-C*H$_2$*-N), 3.64-3.01 (24H, m, N(C*H$_2$*-CH$_2$)$_2$), 2.03 (24H, m, N(CH$_2$-C*H$_2$*)$_2$). **^{13}C{^{1}H} NMR (101MHz, D_2O) δ (ppm):** 150.4 (P-O-*C*^{Ar}), 131.9 (^{Ar}CH), 128.1 (^{Ar}*C*-CH$_2$), 121.5 (^{Ar}CH), 54.9 (N(*C*H$_2$-CH$_2$)$_2$), 50.9 (^{Ar}C-*C*H$_2$), 49.6 (NH-*C*H$_2$-CH$_2$-N), 42.3 (NH-CH$_2$-*C*H$_2$-N), 22.6 (N(CH$_2$-*C*H$_2$)$_2$). **^{31}P{^{1}H} NMR (162MHz, D_2O) δ (ppm):** 8.63.

Compound AK 71

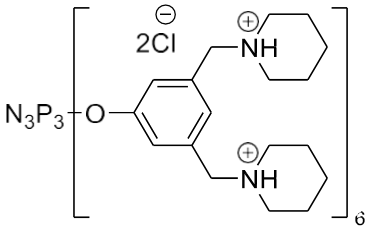

$C_{108}H_{162}N_{15}O_6P_3$, M_r = 1859.50 g/mol. ^{1}H NMR (400MHz, D_2O) δ (ppm): 7.53 (12H, s, $^{Ar}C\underline{H}$), 7.50 (6H, s, $^{Ar}C\underline{H}$), 4.24 (24H, s, $C\underline{H_2}$-N), 3.45-3.18 (24H, m, $N(C\underline{H_2}CH_2)_2CH_2$), 2.97-2.70 (24H, m, $N(C\underline{H_2}CH_2)_2CH_2$), 1.86-1.43 (60H, m, $N(CH_2C\underline{H_2})_2CH_2$, $N(CH_2CH_2)_2C\underline{H_2}$), 1.41-1.17 (12H, m, $N(CH_2CH_2)_2C\underline{H_2}$). **$^{13}C\{^1H\}$ NMR (101MHz, D_2O) δ (ppm):** 150.1 (P-O-$\underline{C}^{Ar}$), 132.1 ($^{Ar}\underline{C}$-CH_2N), 131.9 (^{Ar}CH), 124.9 (^{Ar}CH), 58.9 ($\underline{C}H_2$-N), 53.0 ($N(\underline{C}H_2CH_2)_2CH_2$), 22.6 ($N(CH_2\underline{C}H_2)_2CH_2$), 21.0 ($N(CH_2CH_2)_2\underline{C}H_2$). **$^{31}P\{^1H\}$ NMR (162MHz, D_2O) δ (ppm):** 6.98 (P_3N_3).

Compound AK 72

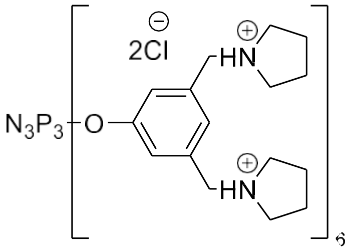

$C_{96}H_{150}Cl_{12}N_{15}O_6P_3$, M_r = 2128.68 g/mol. ^{1}H NMR (400MHz, D_2O) δ (ppm): 7.50 (18H, s, $^{Ar}C\underline{H}$), 4.33 (24H, s, $C\underline{H_2}$-N), 3.67-3.01 (48H, m, $N(C\underline{H_2}CH_2)_2$), 2.15-1.58 (48H, m, $N(CH_2C\underline{H_2})_2$). **$^{13}C\{^1H\}$ NMR (101MHz, D_2O) δ (ppm):** 150.4 (P-O-$\underline{C}^{Ar}$), 133.9 ($^{Ar}\underline{C}$-CH_2N), 129.9 (^{Ar}CH), 123.8 (^{Ar}CH), 56.5 ($\underline{C}H_2$-N), 53.9 ($N(\underline{C}H_2CH_2)_2$), 22.5 ($N(CH_2\underline{C}H_2)_2$). **$^{31}P\{^1H\}$ NMR (162MHz, D_2O) δ (ppm):** 7.04 (P_3N_3).

Compound AK102

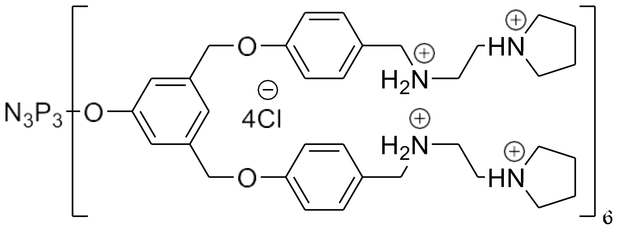

$C_{204}H_{282}Cl_{24}N_{27}O_{18}P_3$, M_r = 4344.39 g/mol. ^{1}H NMR (400MHz, D_2O) δ (ppm): 7.57-7.06 (30H, m, ^{Ar}C*H*), 7.03-6.85 (12H, m, ^{Ar}C*H*), 6.85-6.50 (24H, m, ^{Ar}C*H*), 4.24-4.01 (24H, m, O$C\underline{H_2}$), 3.72-3.00 (120H, m, C$\underline{H_2}$N, NHC$\underline{H_2}$C$\underline{H_2}$N, N(C$\underline{H_2}$$CH_2$)$_2$), 2.39-1.75 (48H, m, N(C$\underline{H_2}$$CH_2$)$_2$). **^{13}C{^{1}H} NMR (101MHz, D_2O) δ (ppm):** 158.7 (^{Ar}*C*-OCH_2), 150.1 (P-O-*C*^{Ar}), 139.0 (^{Ar}*C*-CH_2O), 131.6 (^{Ar}CH), 123.5 (^{Ar}CH), 122.9 (^{Ar}*C*-CH_2N), 119.0 (^{Ar}CH), 115.4 (^{Ar}CH), 68.7 (O*C*H_2), 54.8 (*C*H_2N), 51.1 (N(*C*$H_2$$CH_2$)$_2$), 49.7 (NH*C*$H_2$$CH_2$N), 42.1 (NH$CH_2$*C*$H_2$N), 29.6 (N($CH_2$*C*$H_2$)$_2$). **^{31}P{^{1}H} NMR (162MHz, D_2O) δ (ppm):** 8.58 (P_3N_3).

Compound AK 116

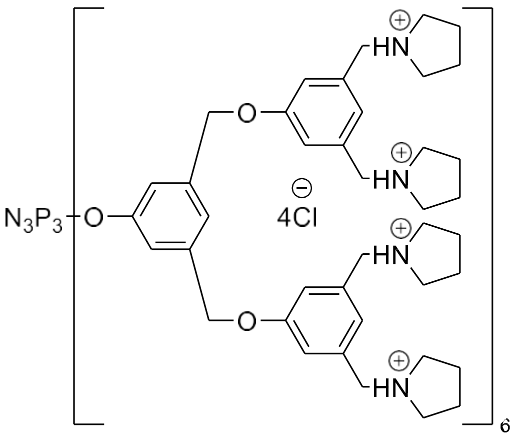

$C_{240}H_{342}Cl_{24}N_{27}O_{18}P_3$, M_r = 4837.27 g/mol. ^{1}H NMR (400MHz, D_2O) δ (ppm): 7.43 (6H, s, ^{Ar}C*H*), 7.14 (12H, s, ^{Ar}C*H*), 7.12 (24H, s, ^{Ar}C*H*), 7.09 (12H, s, ^{Ar}C*H*), 4.99 (24H, s, O$C\underline{H_2}$),

4.18 (48H, C$\underline{H_2}$N), 3.62-3.14 (48H, m, N(C$\underline{H_2}$CH$_2$)$_2$), 3.14-2.83 (48H, m, N(C$\underline{H_2}$CH$_2$)$_2$), 2.25-1.56 (96H, m, N(CH$_2$C$\underline{H_2}$)$_2$). **^{13}C{^{1}H} NMR (101MHz, D$_2$O) δ (ppm):** 158.6 (^{Ar}$\underline{C}$-OCH$_2$), 150.3 (P-O-$\underline{C}^{Ar}$), 139.2 (^{Ar}$\underline{C}$-CH$_2$), 133.2 (^{Ar}$\underline{C}$-CH$_2$), 124.6 (^{Ar}CH), 123.8 (^{Ar}CH), 118.7 (^{Ar}CH), 118.1 (^{Ar}CH), 69.1 (O$\underline{C}$H$_2$-C^{Ar}), 57.1 ($\underline{C}$H$_2$N), 53.7 (N($\underline{C}$H$_2$CH$_2$)$_2$), 22.5 (N(CH$_2$$\underline{C}H_2$)$_2$). **^{31}P{^{1}H} NMR (162MHz, D$_2$O) δ (ppm):** 7.63 (P$_3$N$_3$).

Compound AK 120

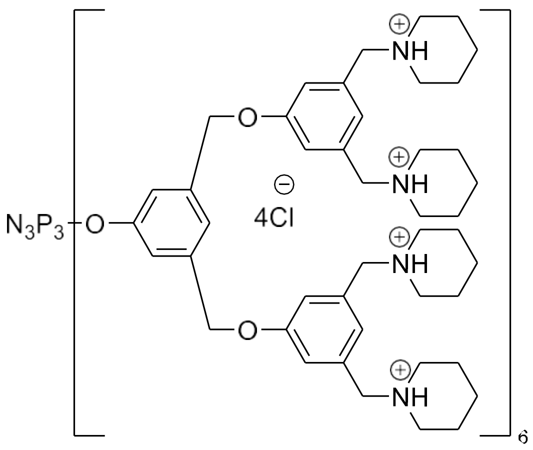

$C_{264}H_{390}Cl_{24}N_{27}O_{18}P_3$, M$_r$ = 5173.92 g/mol. ^{1}H NMR (400MHz, D$_2$O) δ (ppm): 7.45 (6H, s, ^{Ar}C$\underline{H}$), 7.16 (24H, s, ^{Ar}C$\underline{H}$), 7.14 (12H, s, ^{Ar}C$\underline{H}$), 7.10 (12H, s, ^{Ar}C$\underline{H}$), 5.02 (24H, s, OC$\underline{H_2}$), 4.10 (48H, C$\underline{H_2}$N), 3.36-3.16 (48H, m, N(C$\underline{H_2}$CH$_2$)$_2$CH$_2$), 3.04-2.64 (48H, m, N(C$\underline{H_2}$CH$_2$)$_2$CH$_2$), 2.01-1.45 (120H, m, N(CH$_2$C$\underline{H_2}$)$_2$C$\underline{H_2}$), 1.41-1.08 (24H, m, N(CH$_2$CH$_2$)$_2$C$\underline{H_2}$). **^{13}C{^{1}H} NMR (101MHz, D$_2$O) δ (ppm):** 158.4 (^{Ar}$\underline{C}$-OCH$_2$), 150.4 (P-O-$\underline{C}^{Ar}$), 139.3 (^{Ar}$\underline{C}$-CH$_2$), 131.4 (^{Ar}$\underline{C}$-CH$_2$), 126.6 (^{Ar}CH), 123.7 (^{Ar}CH), 119.2 (^{Ar}CH), 118.5 (^{Ar}CH), 69.1 (O$\underline{C}$H$_2$-C^{Ar}), 59.5 ($\underline{C}$H$_2$N), 52.9 (N($\underline{C}$H$_2$CH$_2$)$_2$CH$_2$), 22.6 (N(CH$_2$$\underline{C}H_2$)$_2CH_2$), 21.0 (N(CH$_2CH_2$)$_2$$\underline{C}H_2$). **^{31}P{^{1}H} NMR (162MHz, D$_2$O) δ (ppm):** 7.40 (P$_3$N$_3$).

3. Molecular Modeling

Materials & Methods

All-atom three-dimensional models of siRNA with sequences sense 5' 3' UACUCAGAUCGUGUCACGUdTdT and antisense 3' 5' dTdTAUGAGUCUAGCACAGUGCA were constructed by both, RNA Biopolymer builder provided in Maestro (double-stranded A- or B-RNA) and webserver Vfold3D. More specifically, the sequences submitted to Vfold3D was modified to CCUUUACUCAGAUCGUGUCACGUUUCCCCUUACGUGACACGAUCUGAGUAUUC C as a single sequence composed of overhanging tails, sense, antisense, and a transitory loop region while providing the expected base pairings as 2D structure. As control, submission of this sequence to Vfold2D resulted in the expected base pairings for the most probable 2D structure. Superfluous tails and loop were subsequently deleted from the 3D model, manual corrections applied to transform U to dT, construct proper 3' and 5' ends, and adjust residue numbering and chain names. All siRNA models were compared by superposition to siRNA of PDB entries 1R9F and 2F8S, or 17-mer RNA duplex of 4KYY. The Vfold3D model comparing best to the experimentally determined models in terms of helical diameter, rotational angle, and translational distance along the central axis, was selected for further modeling. Thus, it was subjected to MD simulations in Desmond on NVIDIA V100 graphical processing units for 1µs as NPT ensemble at 300K and 1atm, applying force field OPLS3e, infinite boundary conditions, Nose-Hoover chain thermostat, and Martyna-Tobias-Klein barostat, after neutralizing the system at pH 7 with potassium ions (K^+), constructing an explicit SPC water model including 0.154 M potassium chloride (KCl), and standard pre-equilibration. Energy values and atomic coordinates were recorded every 25 and 250 ps, respectively. The system was well equilibrated in the production phase as evinced by Simulation Quality Analysis. The trajectories were analyzed by Simulation Event Analysis tool and command line scripts. The recorded frames were subjected to Desmond Trajectory Clustering provided in Maestro with settings "Frequency 10" and "Use up to 40 clusters" providing 36 clusters populated with 2 to 23 members. To identify four most divers RNA conformations at the 5' and 3' ends as the central regions of the models were very similar, the 36 centromeres were hierarchically clustered with the conformer_cluster python script provided in the Schrödinger package applying linkage method "average" on root-mean-square deviation (rmsd) of the atomic coordinates of all phosphate atoms. The representative of the first cluster serving as reference was centered at the origin of the coordinate system, its central helical axis aligned along axis z, and the other three

centromeres superposed. The four siRNA models were prepared for molecular docking of models of AK35 in Glide by deleting all water molecules and ions prior to grid calculations. Five grids were calculated on model 1 centered at the origin or shifted along axis z to positions ± 20 and ± 40 Å. Six additional grids were calculated on models 2 to 4, at z positions ± 40 Å to capture the conformational diversity at the 5' and 3' ends for interactions with dendrimer. Inner and outer box dimensions were 20 and 56 Å, respectively, in x, y, and z; else standard settings were applied and no constraints, rotatable groups or excluded volumes defined. Docking was performed in XGlide with standard settings by providing the precalculated grids and AK35 models of all three protonation states in the lowest energy conformation issued from the conformational searches. Low energy conformations of fully protonated models of AK71, AK72, and AK102 (only tertiary amines protonated) were constructed and subjected to MD simulations by the same procedure. Models of fully protonated AK71 and AK72 were also docked by XGlide to the precalculated grids.

Stability of AK35/siRNA dendriplexes was studied by two NPT molecular dynamics simulations of 1µs at 1atm and 300K with explicit solvent including 154 mM KCl and neutralized by 71 Cl^-. The dendriplex was constructed stepwise: three of the previously obtained poses of AK35 with three secondary amines protonated that did not interfere sterically were combined with siRNA in one model, grids at positions 0 Å, 20 Å, and -40 Å along axis z were calculated with inner box and outer box dimensions of 20 Å and 56 Å, respectively, and AK35 with three protonated secondary amines docked by xglide. The new two lowest energy poses were added to form an AK35/siRNA dendriplex of 5:1 stoichiometry. On this model novel grids with box dimensions of 30 Å and 66 Å were calculated at z axis positions of 0 and ± 30 Å, AK35 docked to the dendriplex, and the lowest energy poses integrated in the model. This scheme of novel grid calculation, docking, and increasing dendriplex size was repeated, once more with box dimensions of 30 Å and 66 Å at z axis positions of 0 and ± 30 Å; and subsequently twice with box dimensions of 40 Å and 76 Å at z axis positions of 0 and ± 40 Å, until no novel poses were obtained. The final dendriplex of 13:1 stoichiometry was prepared for molecular dynamics simulations in a cube of 123 Å edge length.

Results

Conformational space, surface properties, and molecular dynamics (MD) of AK35 were studied by classical force field methods on three-dimensional all-atom models in three protonation states. Indeed, AK35 includes six tertiary and six secondary amines in the branches. The tertiary amines were always modeled as protonated while the secondary amines were either all

protonated, deprotonated, or three of six homogeneously distributed around the N_3P_3 core protonated. Assuming, that the precise charge distribution statistically tends to repartition dynamically and uniformly throughout the molecular volume of AK35 and that its overall conformational dynamics and shape are at the utmost marginally affected by the different combinations of charge repartition in the partial protonated model, we limited the study to one combination of partial protonation. Conformational searches of the three models issued lowest potential energy conformations which evinced solvent-accessible surface areas (SASA) with very dominantly positive or neutral potential energy (Figure S1). These SASA arose to over 85% from the branches in models with all or three of six protonated secondary amines, while solvent exposed regions of the N_3P_3 core contributed the remaining less than 15% of total SASA with a slightly negative potential energy (Figure S1, A and B). AK35 modelled with secondary amines deprotonated showed 100% SASA provided by the branches which entirely shielded the core from solvent due to less electrostatic repulsion compared to the other two protonation states (Figure S1C).

MD of these three models were simulated for 1µs with an explicit water model including 0.154 M sodium chloride (NaCl) under standard conditions in a well equilibrated production phase. As previously reported for chemically closely related dendrimers AK35 appeared as a conformationally highly mobile molecule in all three protonation states investigated of which the branches typically interacted electrostatically with several chlorine ions (Cl^-) and hydrogen bonded to water molecules [1]. Cl^- interacted with up to four protonated amines in the models with charged secondary amines, else typically with one or two counter ionic groups. The cationic centers of the branches interacted with up to three Cl^-. Sodium ions (Na^+) seldom diffused closer than 3 Å to the dendrimers, only 0.2 to 0.3% of recorded frames of either MD simulation showed the cation near the dendrimers. Intra-dendrimer interactions occurred frequently as π-π-stacking or π-cationic arrangements between branches on either the same or opposite sides of the N_3P_3 core. The N_3P_3 cores typically interacted with one to three water molecules (Figures S3, S6, and S9). However, the different electrostatic repulsion of the branches due to the three protonation states of the models induced slight differences in molecular shape and interaction with bulk water. The radial distribution function of water around the N_3P_3 cores characterized a shorter dimension of the dendrimers of 8 to 8.5 Å while the longer dimension was 15, 16, or 14 Å for models with all, three of six, or no protonated secondary amines (Figures S2, S5, and S8). Accordingly, the radii of gyration (r_{Gyr}) decreased from 8.6 over 8.0 to 7.5 Å with decreasing protonation (Figures S3, S6, and S9). The shrinking

in size is also reflected in the number of hydrogen bonds with bulk water: The branches of entirely protonated AK35 typically formed 10 to 20 hydrogen bonds to water molecules, intermediary protonation reduced hydrogen bonding of the branches to 8 to 16 water molecules, and no protonation of secondary amines typically resulted in 6 to 11 hydrogen bonds (Figures S3, S6, and S9). Increased shielding of the N_3P_3 core from solvent by reduced protonation of the branches was also evinced: While the core of all or intermediary protonated AK35 models frequently formed more than three hydrogen bonds to water molecules, such number of interactions was less often observed for least protonated AK35 (Figures S3, S6, and S9).

The lowest energy conformation identified for fully protonated AK35 suggested maximization of distance between the tertiary amines such that its global shape resembled a disk with a rim thicker than the center (Figure S1A). This shape associated with branches extending rather perpendicularly from the N_3P_3 cycle, frequently occurred during the MD simulation, thus giving rise to maximum Max1 of the radial distribution function of the tertiary amines around 19 Å, while the smaller maximum Max2 around 9 Å was due to branches from above and below the N_3P_3 core tilting towards the core plane and approaching each other (Figure S4C). The lowest energy conformation identified for intermediary protonated AK35 assumed a global shape that reminded of a half-sphere as some of the branches bended back towards the N_3P_3 core (Figure S1B). This global shape also dominating in the MD simulation, gave rise to a maximum around 17 Å in the radial distribution function of the tertiary amines above and below the N_3P_3 cycle (Figure S7C). Thus, branches bending back clearly reduced the distance between tertiary amines above and below the plane of the core cycle compared to fully protonated AK35. Also, there was no second maximum observed, rather a faint shoulder around 11 Å due to back-curling branch conformations of intermediary protonated AK35 (Figure S7C). The almost spherical shape of AK35 modeled without protonation of secondary amines suggested by the lowest energy conformation identified in the conformational search, was also reflected in the radial distribution function of the tertiary amines above and below the N_3P_3 core (Figures S1C and S10C): the maximum was observed around 14 Å evincing the tendency of the branches to bend back to the N_3P_3 center and shield it from solvent. This tendency was in line with a higher number of instances in which no hydrogen bonds of core and water molecules were detected as well as with less hydrogen bonding of the branches to water compared to fully or intermediary protonated AK35 (Figures S9 *versus* S6 and S3). To summarize in one sentence: molecular modeling suggested that increasing protonation of AK35 modifies its shape and increases its

size in the largest dimension as well as hydration of the branches and solvent access to the N_3P_3 core.

Models of fully protonated AK71 and AK72 or AK102 with only tertiary amines protonated behaved similarly in MD simulations compared to AK35. AK71 and AK72 showed r_{Gyr} of 8.1 ± 0.1 Å and 7.8 ± 0.1 Å, respectively. Their longest end-to-end distances ($d_{end-end}$) were 14.8 ± 4.5 Å and 12.5 ± 4.1 Å, respectively (Figure S13). AK102 had slightly larger dimensions with an r_{Gyr} of 10.1 ± 0.3 Å and $d_{end-end}$ of 18.6 ± 5.7 Å (Figure S13).

To study the interactions of phosphorus dendrimers with their target, models of siRNA with sequences sense 5' 3' UACUCAGAUCGUGUCACGUdTdT (strand A) and antisense 3' 5' dTdTAUGAGUCUAGCACAGUGCA (strand B) were obtained either by constructing double-stranded siRNA with canonical A- or B-fold helical parameters or by the knowledge-based method implemented in Vfold3D. The resulting models were compared to experimentally determined models of siRNA or double-stranded RNA (dsRNA). The model obtained by Vfold3D compared best to the experimental models in terms of helical parameters and was therefore subjected to 1µs of MD simulations in explicit aqueous solution including 0.154 M potassium chloride (KCl) to challenge its stability and obtain conformations for molecular docking of phosphorus dendrimer models. Potassium (K^+) and KCl were chosen for neutralization and provision of supplementary ions as K^+ is the most abundant (earth-)alkaline cation in the cytosol of mammalian cells while rather forming an "ionic atmosphere" around RNA with transitory interactions instead of inducing specific RNA conformations or folds. The siRNA model showed and average volume (V_{av}) of 182.4 nm^3 with a standard deviation (std) of ± 0.6 nm^3 with r_{Gyr} of 2.0 ± 0.07 nm, and $d_{end-end}$ of 5.8 ± 0.4 nm (Figure S13). Thus, although being a small RNA macromolecule, it was still larger than the phosphorus dendrimers of this study.

Throughout the simulation, the central region of the double-stranded helix was stable in terms of base pairing and helical parameters while losses of canonical pairing were observed at the 5' and 3'ends. The pair $U19^A/A21^B$ at the 3' end of strand A rapidly lost canonical hydrogen bonding, however fluctuating π-stacking and non-canonical hydrogen bonds implicating $U19^A$ to $dT21^A$ and $A21^B$ stabilized the siRNA end such that the preceding $G18^A/C20^B$ pair conserved canonical interactions throughout the simulation. The pair $U1^A/A3^B$ at the 5' end of strand A conserved canonical hydrogen bonding for almost 200 ns. Once canonical pairing leaving place to non-canonical interactions, this siRNA end was less efficiently stabilized than the other end

such that during the last 75 ns of the simulation the subsequent pair $A2^A/U4^B$ also lost canonical pairing. Observation of higher conformational flexibility of the 5' and 3' ends compared to the central regions of dsRNA is typical in MD simulations. In function of the scope of such studies, structural and conformational analyses may have to focus on the central stretch of dsRNA models. Therefore, we did not impute the occurrence of the observed non-canonical conformations to the $(dT)_2$ overhangs although they influenced the structural dynamics by both, intra- and intermolecular interactions with siRNA and solvent compared to dsRNA with blunt ends. Assuming AK35, AK71 and AK72 might bind to its target in the central regions and at the termini, four representatives were identified by clustering of the recorded MD conformations to capture structural variability and prepared for automated docking of AK71, AK72, and AK35 models in the three protonation states studied previously.

Parameters of the automated docking procedure were chosen to least bias the results to a particular region or favor one type of interactions over others. Thus, the centers of the grid boxes calculated prior to automated placement of AK35 models were defined at the center of the siRNA model and shifted by ±20 Å along the helical axis to explore interactions with the central region. To specifically evaluate possible interactions at the 5' and 3' siRNA ends, the box centers were translated by ±40 Å along the helical axis. Inner and outer cubic boxes of 20 and 56 Å edge size, respectively, ascertained generous overlap of the outer grid boxes and that no gaps between inner boxes disfavored certain siRNA regions in the docking study. AK35 docked to the central regions of target siRNA always in the major groove, no matter which of the three protonation states was considered. In these poses the N_3P_3 core did not directly interact with siRNA, whilst the branches formed interactions with both strands, A and B (Publication Figure 3). Thereby the dendrimer bridged the phosphate backbones of the RNA strands over the major groove by electrostatic interactions of protonated secondary and tertiary amines. The branches also hydrogen bonded by neutral amines in addition to forming π-π-stacking, π-cation interactions, classical and aromatic hydrogen bonds to RNA bases (main text Figure 3). Thus, the dendrimer smoothly adhered to the inner face of the helical RNA ribbon by forming interactions with all six branches. Docking of the AK35 models to the 5' and 3' ends of the four conformational siRNA representatives revealed a similar picture: all types of hydrogen bonds, electrostatic and π-interactions were observed when the dendrimer embraced the ends of the helical ribbon to interact with phosphate, ribose, and nucleobase units (main text Figure 3 and Figure S11). Interactions were more often formed on the inner major groove face of the RNA ribbon than on the outer minor groove face. While not necessarily all AK35 branches interacted

simultaneously in these poses with the target, direct hydrogen bonding of the N_3P_3 core to a terminal adenine occurred (Figure S11A).

Stability and SASA of AK35/siRNA dendriplexes were explored by two molecular dynamics simulations of 1µs at standard conditions. Already in the construction of the dendriplex with 13:1 stoichiometry it was striking that all AK35 poses targeted the major inner groove only. Once entirely filled, interactions in the minor outer groove were below the energetic threshold of the standard docking parameters, such that no further poses were retained. Simulation quality analysis of the production phases of molecular dynamics that had been preceded by the standard pre-equilibration protocol, revealed an equilibrated system after 50 ns. Early in the simulations individual dendrimers diffused away from and again towards the dendriplex. After 200 ns of simulation the dendriplexes were stable with 10:1 stoichiometry, while three AK35 molecules diffused freely in the solvent phase in both independent calculations. Throughout the entire production phases the major inner grooves were shielded from solvent by AK35 whilst the minor outer grooves remained largely solvent accessible. Thus, the SASAs of AK35/siRNA dendriplexes appeared as two ribbons wrapping side-by-side in a helical fashion around the nucleic acid whereas one ribbon had a positive and the other a negative electrostatic potential (Figure S16).

Discussion

Aminoglycoside antibiotics bind in the large groove of procaryotic, human, or protozoal ribosomal decoding site RNAs. The majority of interactions per antibiotic is provided by direct or water-mediated hydrogen bonds to carbonyl-oxygens and amino groups of the nucleobases. Typically, one to three hydrogen bonds to the phosphate backbone complete the interaction schemes of the antibiotics [2-6].

The structure-activity relationships of the aminoglycosides distinctly concern their biological mechanisms of action, affinity, and selectivity for prokaryotic, protozoal, or eukaryotic ribosomal RNA A sites. While apramycin, paromomycin, and gentamycin efficiently inhibit translation elongation by binding to prokaryotic ribosomal RNA A site, apramycin assumes distinct binding mode and molecular interaction mechanism that cause only limited miscoding compared to the other two [7]. Apramycin also stabilizes either “on” or “off” decoding conformations when binding to either bacterial or human ribosomal A sites, accordingly inhibition of translation differs by three orders of magnitude; binding affinity of related 2-deoxystreptamine aminoglycosides is not correlated with inhibition of translation in eukaryotic

or prokaryotic systems. NB33 stands out as it differentially binds to “off” state compared to “on” state decoding conformation of human ribosomal A site like apramycin while it is a 10-fold better inhibitor of translation in the eukaryotic, although 22 times weaker in the prokaryotic system compared to apramycin. The exceptional conformational flexibility of NB33 allowing for specific RNA interactions in the human system is recognized as the driving force for its unusual biological activity profile [4].

Thus, aminoglycosides extensively evince that binding mode, affinity, and biological activity are independently ruled by structure and physicochemical properties of RNA-interacting agents. Phosphorous dendrimers show similar subtle differences in the various biological assays, thereby conforming to the aminoglycoside paradigm.

While these small molecules locate and interact preferentially in the major groove of dsRNA with both, nucleobases and phosphates of the RNA backbone, interaction schemes of dsRNA with proteins as for example p19 of tombusvirus and transactivation response RNA binding protein (TRBP2) differ. These proteins wrap around the outer face of the dsRNA ribbon to predominantly interact with the phosphate backbone and ribose units, either directly or mediated by water molecules. At the 5’ and 3’ end either blunt or with overhang, they may form direct interactions with the nucleobases. For instance, p19 forms a triple layered parallel π-stacking by Trp39 and Arg43 with $G19^{C}$ while TRBP only forms one hydrogen bond to a nucleobase from the minor groove face in the central region of the complex: Ala187 and $G8^{B}$. *Aquifex aeolicus* Argonaute (*Aa*Ago) binds to the two-nucleotide overhang of 22-mer and 26-mer siRNA at one 3’-end by specific interactions which are proposed to be of biological relevance. U21 and U22 of the overhang are splayed off the dsRNA helix to parallel π-stack with Tyr119 and hydrogen bond to the backbone nitrogen of Asp191, respectively, by their nucleobases. Their backbone phosphates form ion pairs with Lys256 and Lys186/Arg162, respectively. At the other siRNA end only non-specific packing presumably without biological relevance is observed. Crystal structures of *Thermus thermophilus* Argonaute (*Tt*Ago) in ternary complexes with guide-DNA and target-RNA evince dsDRNA hybrid in A-form, with the protein, again, wrapping around the outer face of the helix and interacting with the phosphate-ribose backbone or nucleobases from the side of the minor groove. For instance, Lys618, Arg574, Lys575, Ser576, and Lys664 form hydrogen bonds or ion pairs with the RNA phosphates of A7’, C8’, U10’, and A11’ and at the 5’-end the aromatic systems of RNA C18’ and His445 π-stack while at the 3’-end Pro44 packs on the nucleobase of RNA A4’ (pdb entry 3HK2).

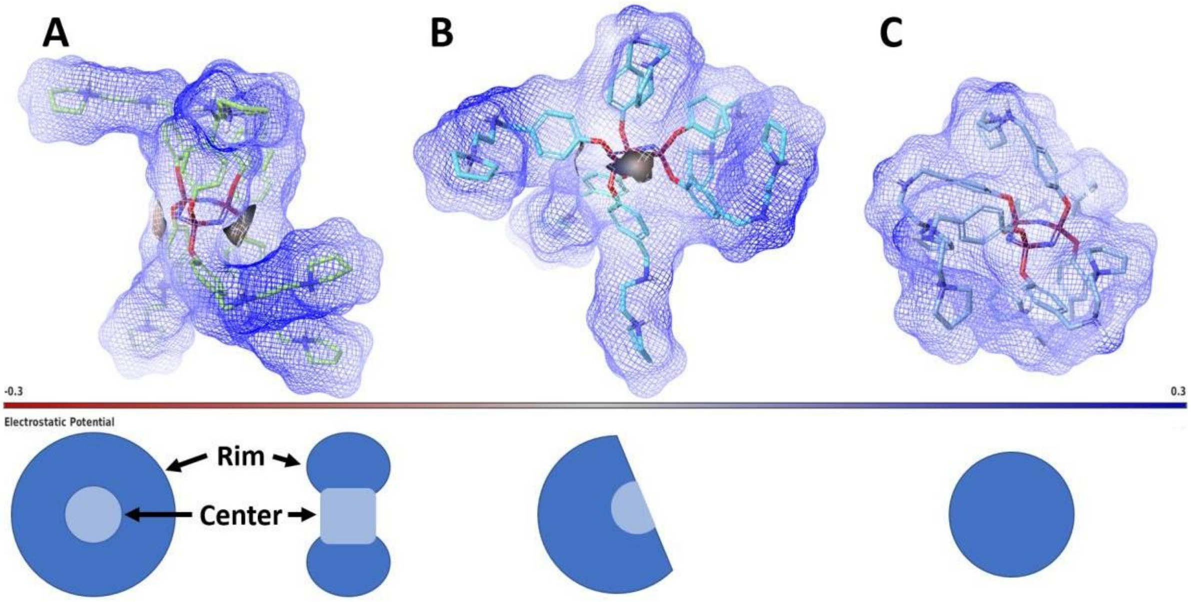

Figure S1: Three-dimensional models of AK35 in three different protonation states. All secondary amines protonated (A), only 3 of 6 secondary amines protonated (B), no secondary amine protonated (C), tertiary amines are always protonated. AK35 presents a solvent-accessible surface area (SASA) with very dominantly positive or neutral potential energy arising to over 85% from the branches in A and B, to a 100% in C. Very localized spots of less than 15% of the entire surface have a slightly negative potential energy arising from solvent exposed regions of the N_3P_3 core in A and B. Branch conformations in C entirely shield the core from solvent. The models are the lowest energy conformations issued from conformational searches applying an implicit solvent model. SASA arising from the entire dendrimer or N_3P_3 core only are depicted as mesh or solid, respectively; color ramp by potential energy from -0.3 in red over neutral in white to 0.3 in blue. The chemical structure of the dendrimers is depicted as tubes with oxygen, nitrogen, phosphor, and hydrogen atoms indicated in red, blue, purple, and white, respectively; carbon atoms are indicated in green or light blue shades; nonpolar hydrogen atoms are not represented for clarity. The sketches at the bottom symbolize simplified general shapes of AK35: facial view and sagittal cut of edge view (A), sagittal cut of edge view (B), facial view (C) with N_3P_3 core and branches symbolized in light and dark blue, respectively.

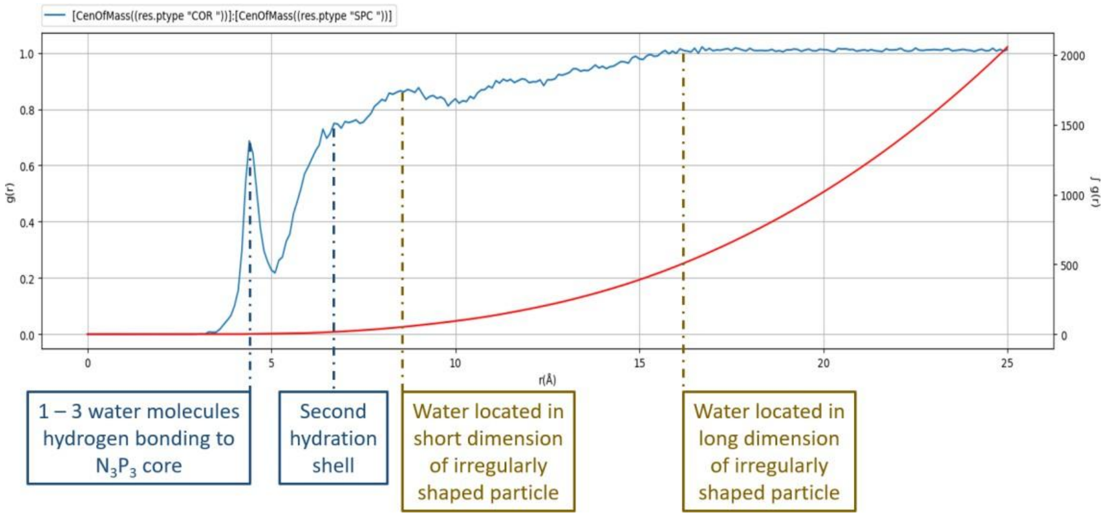

Figure S2: AK35 – all secondary amines protonated: Radial distribution function of water around N_3P_3 core. The molecular dynamics simulation of the dendrimer evinces an irregularly shaped nanoparticle of changing shape with shorter and longer dimensions of about 8.5 and 16 Å, respectively, as defined by the distribution of bulk water. The maximum of the curve at about 4.5 Å arises from hydrogen bonds that the N_3P_3 core forms with water molecules (Figure S3). The peaks of first and second hydration shells of the core, as well as the shorter and longer dendrimer dimensions fit well to a radius of gyration of 8.2 to 8.8 Å (Figure S3).

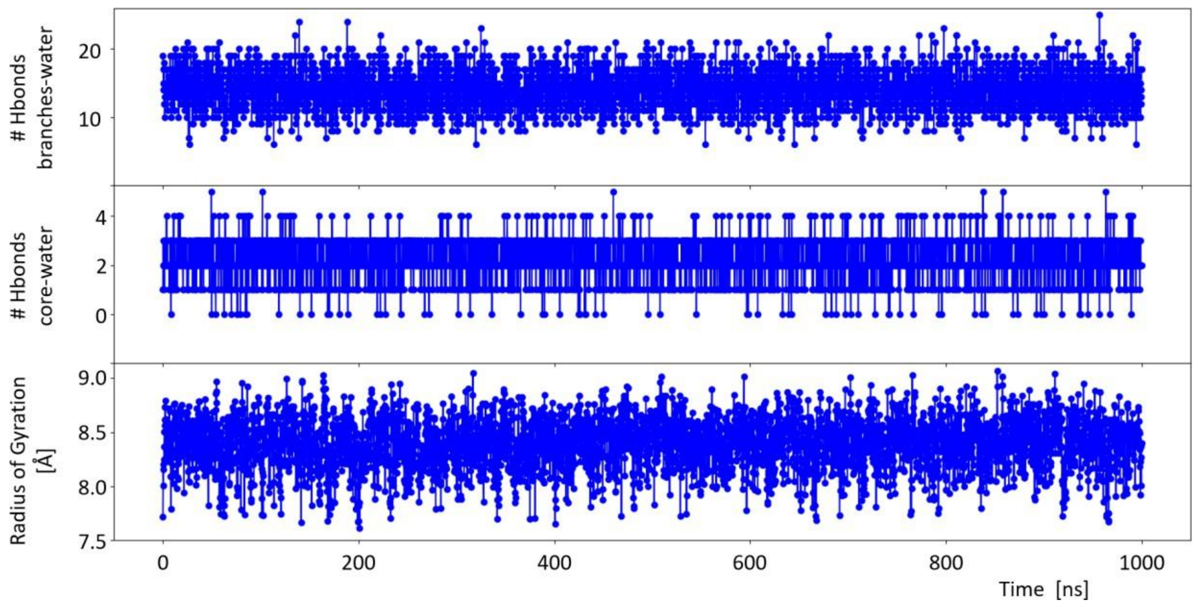

Figure S 3: AK35 – all secondary amines protonated: statistics of hydrogen bonds of branches and N_3P_3 core formed with water molecules and of the radius of gyration *versus* simulation time (top, middle, and bottom, respectively). Branches form typically 10 to 20 hydrogen bonds to water molecules, while the N_3P_3 core hydrogen bonds typically 1 to 3, maximum 5 water molecules. The radius of gyration is typically observed between 8.2 and 8.8 Å.

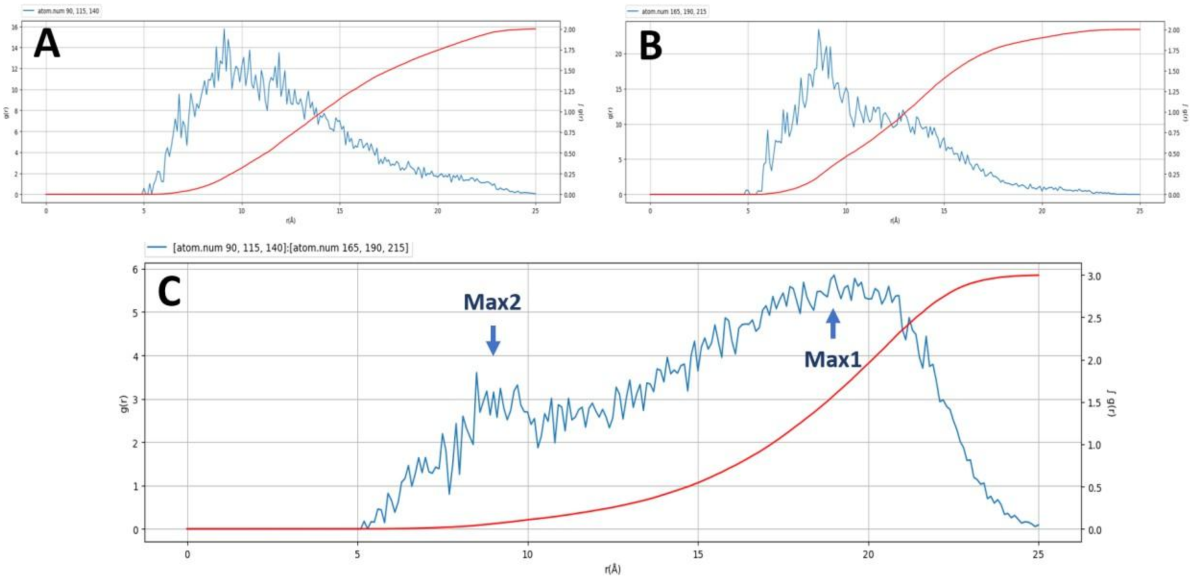

Figure S 4: AK35 – all secondary amines protonated: Radial distribution function of the protonated tertiary nitrogen atoms. Analysis of branches above or below the plane of the N_3P_3 core in A) and B), respectively, of branches above *versus* below in C). The nitrogen atoms tend to be at a distance of 8 to 9 Å in A) and B) as indicated by the maxima of the g(r) curve

(blue), which corresponds to branch conformations that extend roughly perpendicularly to the N_3P_3 plane. These conformations give rise to the maximum Max1 of the g(r) curve around 19 Å in C). The maximum Max2 of g(r) around 9 Å in C) arises from branch conformations that tilt strongly towards the N_3P_3 plane and approach nitrogen atoms of branches above and below the plane. Branches are highly mobile switching frequently from perpendicular to tilted conformations and back, thereby distributing the positively charged nitrogen atoms evenly around the core and optimizing the electrostatic repulsion between the charged groups. Transient interactions of chloride ions and positively charged nitrogen atoms are also observed.

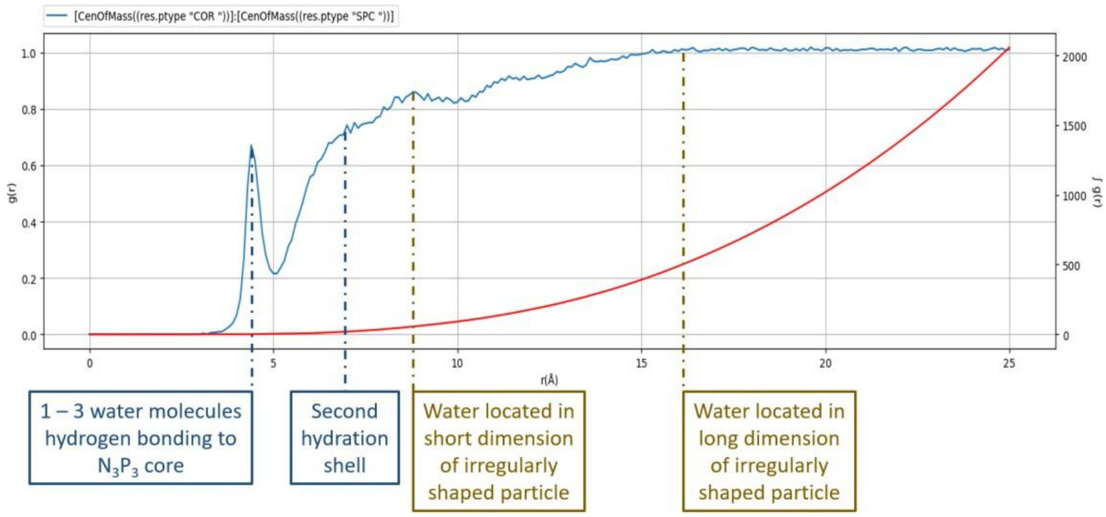

Figure S5: AK35 – 3 of 6 secondary amines protonated: Radial distribution function of water around N_3P_3 core. The molecular dynamics simulation of the dendrimer evinces an irregularly shaped nanoparticle of changing shape with shorter and longer dimensions of about 8.5 and 16 Å, respectively, as defined by the distribution of bulk water. The maximum of the curve at about 4.5 Å arises from hydrogen bonds that the N_3P_3 core forms with exceptionally up to 5 water molecules, typically 1 to 3 (Supplements Figure S6). The peaks of first and second hydration shells of the core, as well as the shorter and longer dendrimer dimensions fit well to a radius of gyration of 7.6 to 8.4 Å (Figure S6).

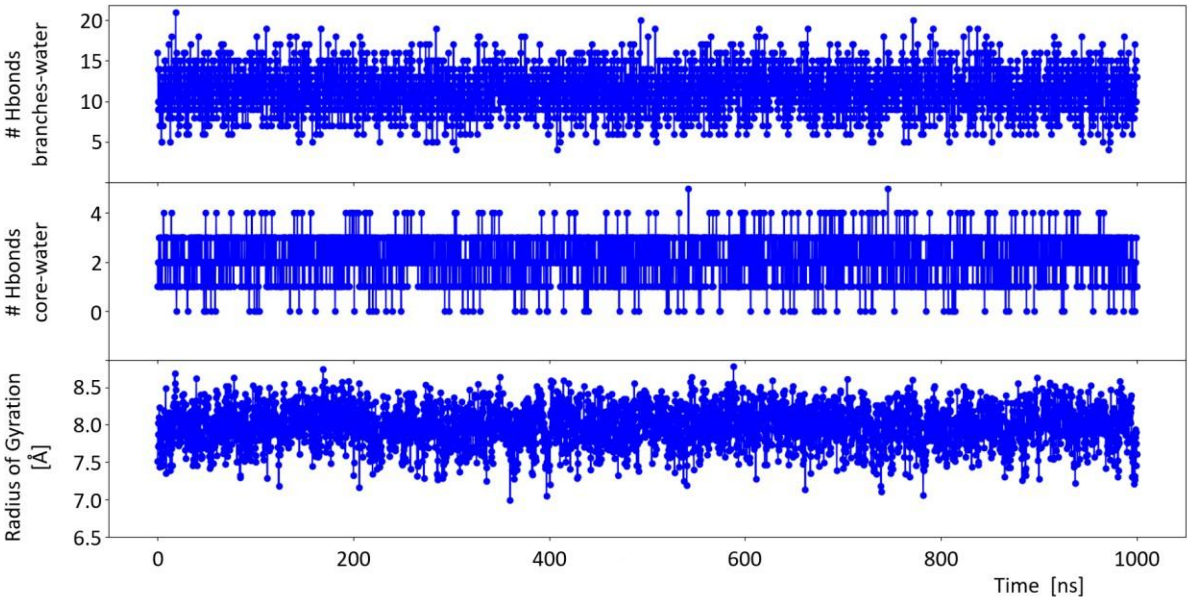

Figure S6: AK35 – 3 of 6 secondary amines protonated: statistics of hydrogen bonds of branches and N_3P_3 core formed with water molecules and of the radius of gyration *versus* simulation time (top, middle, and bottom, respectively). Branches form typically 8 to 16 hydrogen bonds to water molecules, the N_3P_3 core hydrogen bonds typically 1 to 3, only exceptionally 5 water molecules. The radius of gyration fluctuates typically between 7.6 and 8.4 Å.

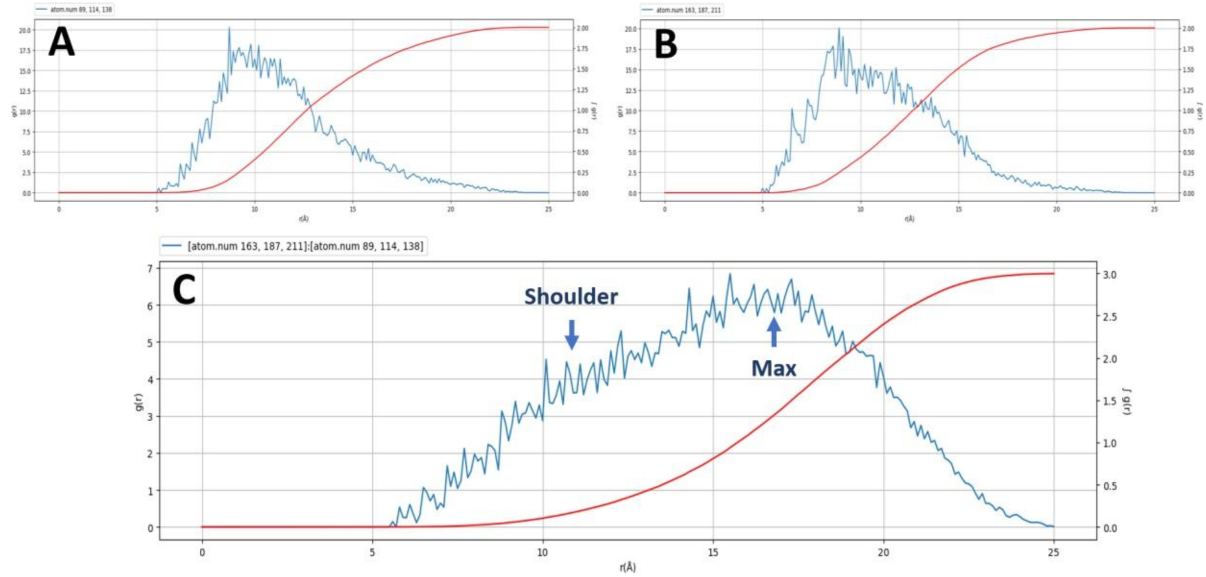

Figure S7: AK35 – 3 of 6 secondary amines protonated: Radial distribution function of the protonated tertiary nitrogen atoms. Analysis of branches above or below the plane of the N_3P_3 core in A) and B), respectively, of branches above *versus* below in C). The nitrogen atoms tend to be at a distance of about 9 Å in A) and B) as indicated by the maxima of the g(r) curve

(blue). Branch conformations that extend roughly perpendicularly to the N_3P_3 plane give rise to the maximum of the g(r) curve around 17 Å in C). Branch conformations that approach the tertiary nitrogen atoms towards the N_3P_3 plane or approach nitrogen atoms of branches above and below the plane accumulate to a shoulder of g(r) around 11 Å in C). Branches are highly mobile switching frequently from perpendicular to tilted conformations and back, thereby distributing the positively charged nitrogen atoms evenly around the core and optimizing the electrostatic repulsion between the charged groups. However, the distinction between conformations roughly perpendicular or tilting towards the N_3P_3 plane is less pronounced compared to AK35 modeled with all secondary amines protonated (Figure S3). Transient interactions of chloride ions and positively charged nitrogen atoms are also observed.

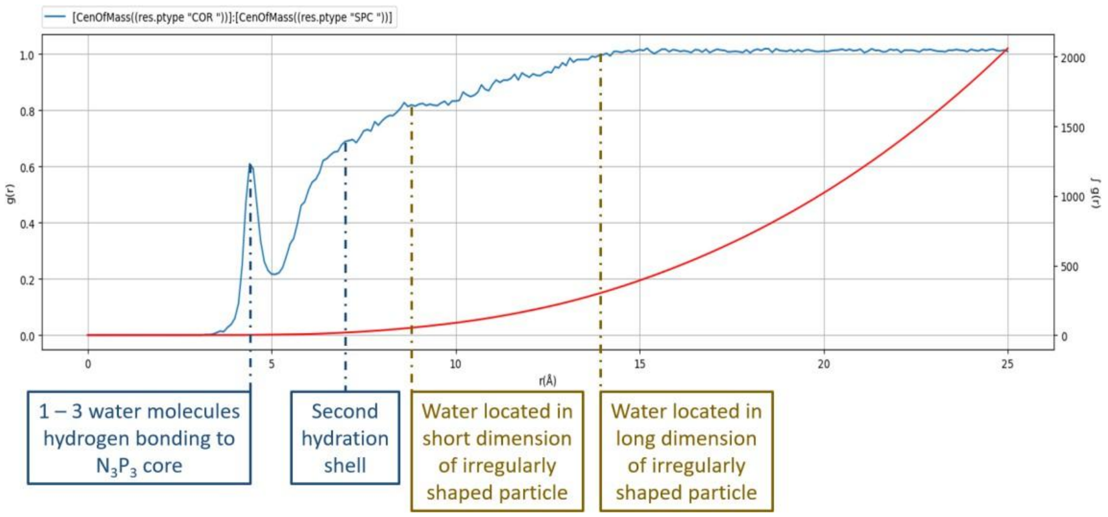

Figure S8: AK35 – no secondary amines protonated: Radial distribution function of water around N_3P_3 core. The molecular dynamics simulation of the dendrimer evinces an irregularly shaped nanoparticle of changing shape with shorter and longer dimensions of about 8.5 and 14 Å, respectively, as defined by the distribution of bulk water. The maximum of the curve at about 4.5 Å arises from hydrogen bonds that the N_3P_3 core forms with exceptionally up to 5 water molecules, typically 1 to 3 (Figure S9). The peaks of first and second hydration shells of the core, as well as the shorter and longer dendrimer dimensions fit well to a radius of gyration of 7.1 to 7.9 Å (Figure S9).

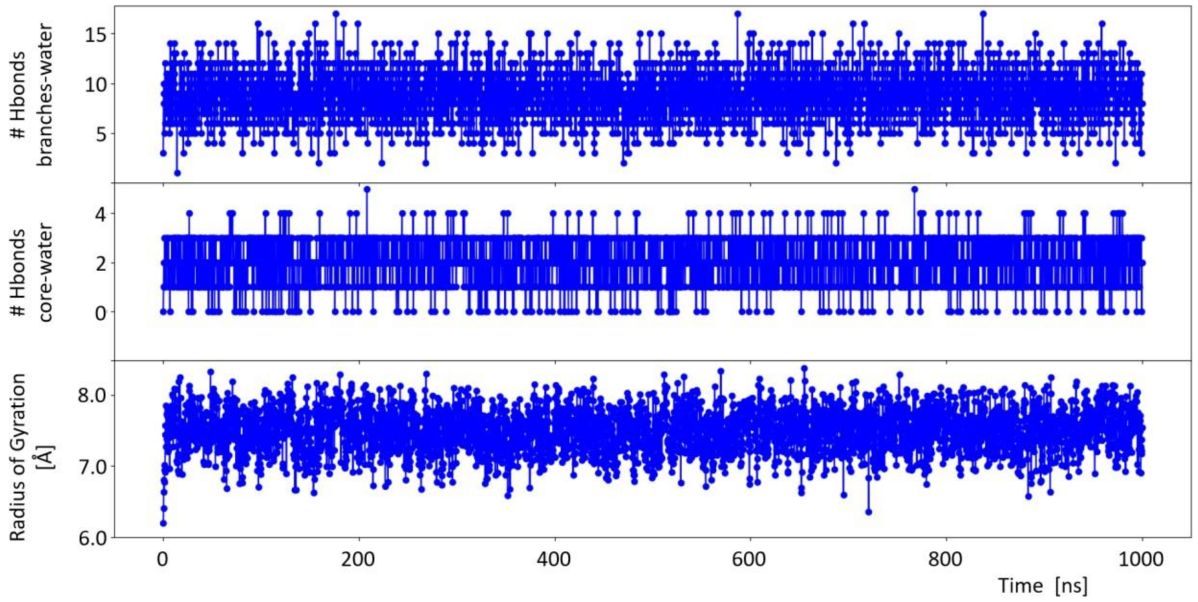

Figure S9: AK35 – no secondary amines protonated: statistics of hydrogen bonds of branches and N_3P_3 core formed with water molecules and of the radius of gyration *versus* simulation time (top, middle, and bottom, respectively). Branches form typically 6 to 11 hydrogen bonds to water molecules, the N_3P_3 core hydrogen bonds typically 1 to 3, only seldom 4 or 5 water molecules. The radius of gyration fluctuates typically between 7.1 and 7.9 Å.

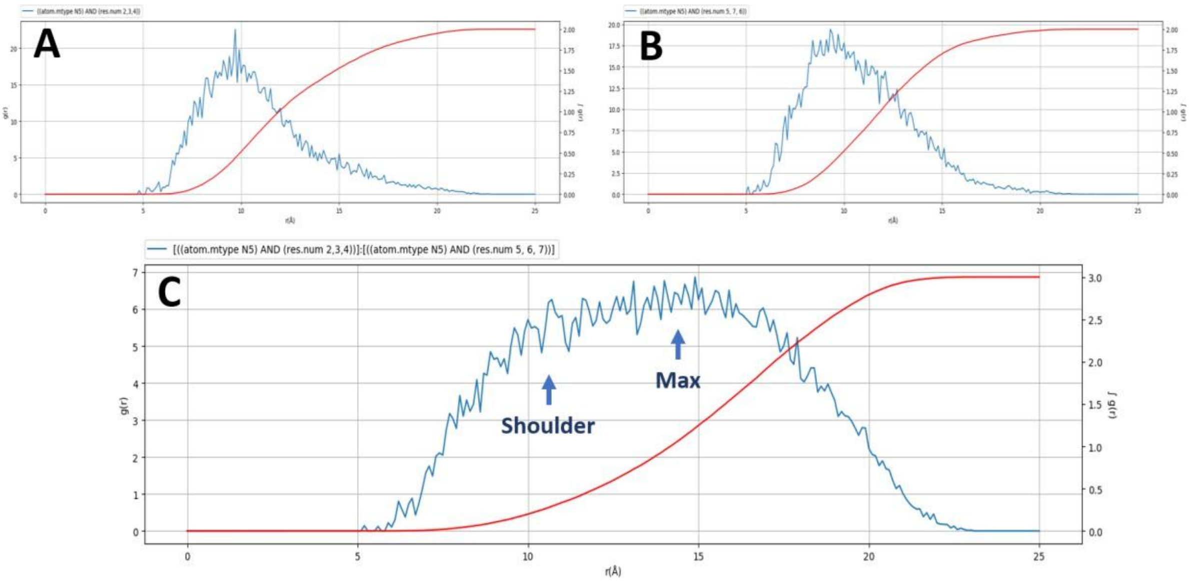

Figure S10: AK35 – no secondary amines protonated: Radial distribution function of the protonated tertiary nitrogen atoms. Analysis of branches above or below the plane of the N_3P_3 core in A) and B), respectively, of branches above *versus* below in C). The nitrogen atoms tend to be at a distance of about 9 Å in A) and B) as indicated by the maxima of the g(r) curve (blue). Branch conformations that extend roughly perpendicularly to the N_3P_3 plane give rise

to the maximum of the g(r) curve around 14 Å in C). Branch conformations that tilt towards the N_3P_3 plane and approach nitrogen atoms of branches above and below the plane accumulate to a faint shoulder of g(r) around 11 Å in C). Branches have highly mobile, easily bending conformations, thereby distributing the positively charged nitrogen atoms evenly around the core and optimizing the electrostatic repulsion between the charged groups. However, the distinction between conformations roughly perpendicular or tilting towards the N_3P_3 plane much less pronounced compared to AK35 modeled with all secondary amines protonated (Figure S3). Transient interactions of chloride ions and positively charged nitrogen atoms are also observed.

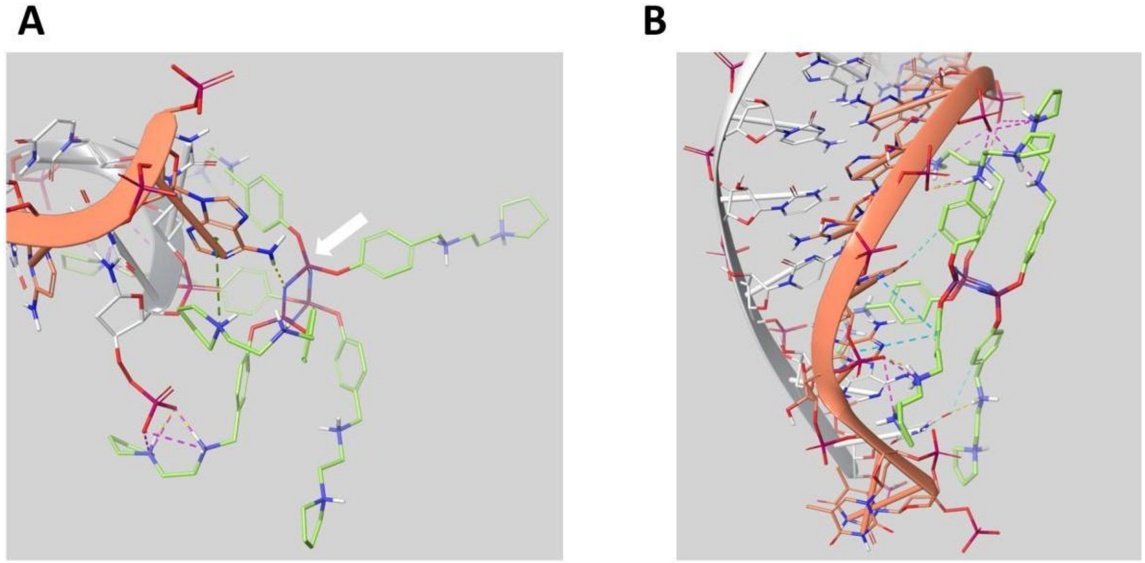

Figure S11: Three-dimensional models of AK35 docked to 3'- and 5'-end of snapshot 3 of siRNA obtained from a MD simulation (A and B). The positively charged secondary and ternary amine groups of the branches form numerous ion pair interactions with the negatively charged phosphate groups of the siRNA. Nevertheless, π-π-stacking, π-cation interactions, classical and aromatic hydrogen bonds between dendrimer branches and RNA bases are also observed in A and B, as well as a hydrogen bond of the N3P3 core with terminal adenine (A).

The chemical structures of dendrimer and siRNA is depicted as tubes with oxygen, nitrogen, phosphor, and hydrogen atoms indicated in red, blue, purple, and white, respectively; carbon atoms are depicted in green, white and brown for AK35 and RNA, respectively; nonpolar hydrogen atoms are not represented for clarity; backbone trace and base orientation of siRNA are visualized by ribbons and sticks. Non-covalent interactions are indicated by dashed lines: salt bridges – magenta; classic and aromatic hydrogen bonds – yellow and light blue, rsp; perpendicular or parallel π-π-stacking – cyan; π-cation – green.

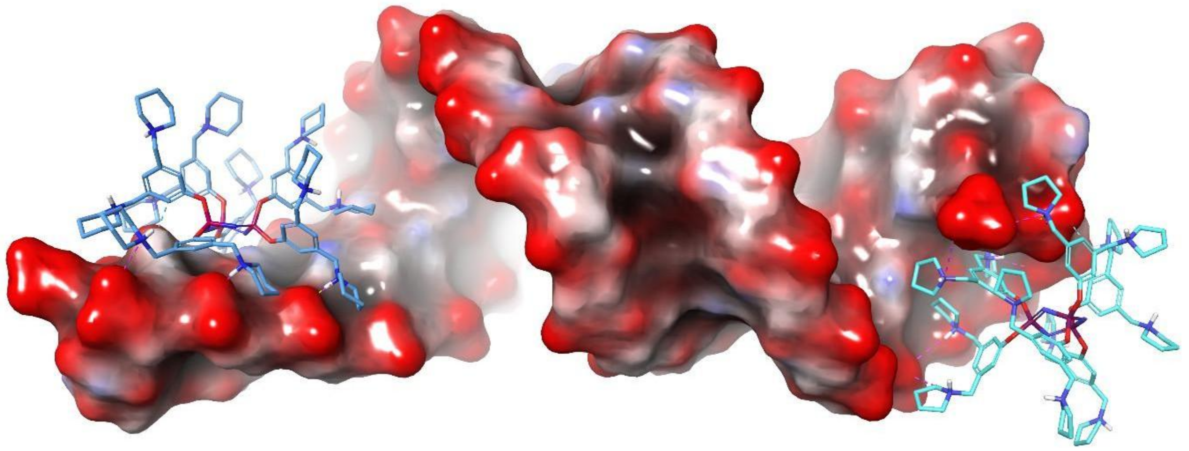

Figure S12: Three-dimensional models of AK71 and AK72 docked to 3'- and 5'-end of snapshot 3 of siRNA obtained from a MD simulation. AK71 and AK72 were only placed in the major inner groove, no poses suggesting capping as AK35 were obtained in either conformation of the siRNA ends. Molecular representation and indication of non-covalent interactions as in publication Figure 3; AK71 and AK72 are depicted with carbon atoms in blue and cyan, respectively.

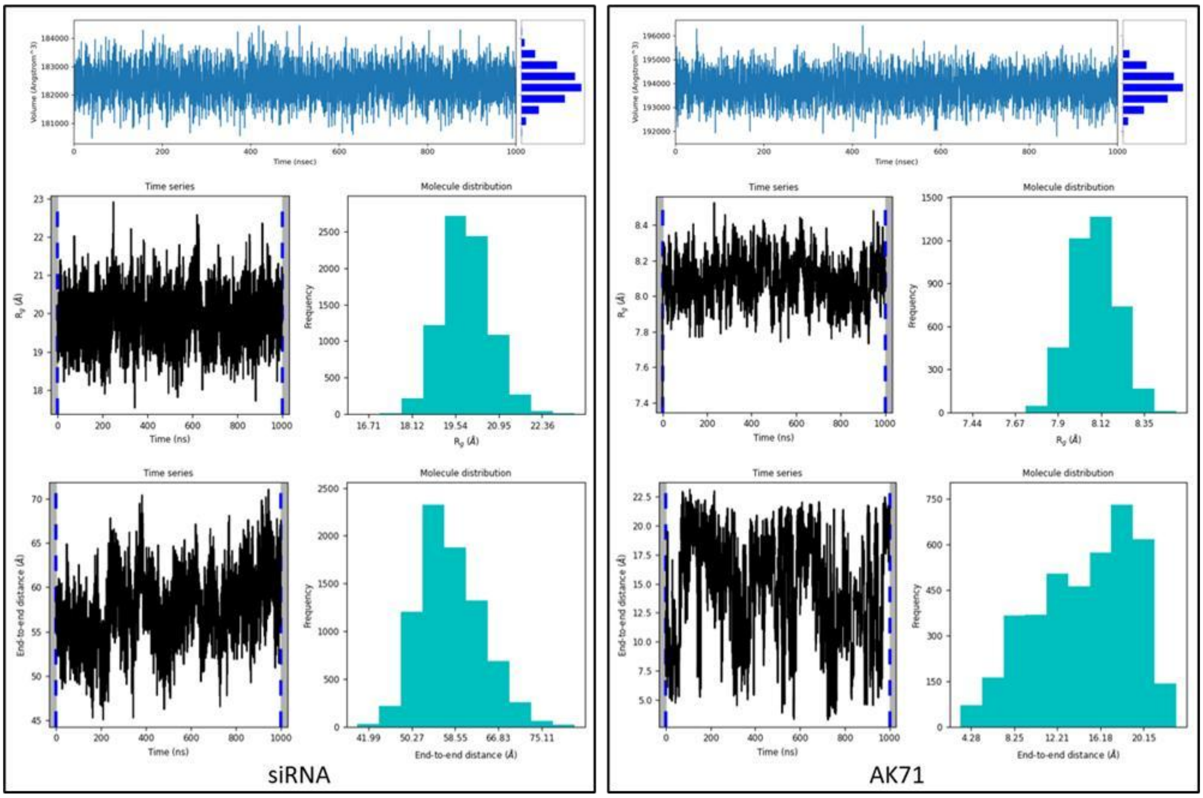

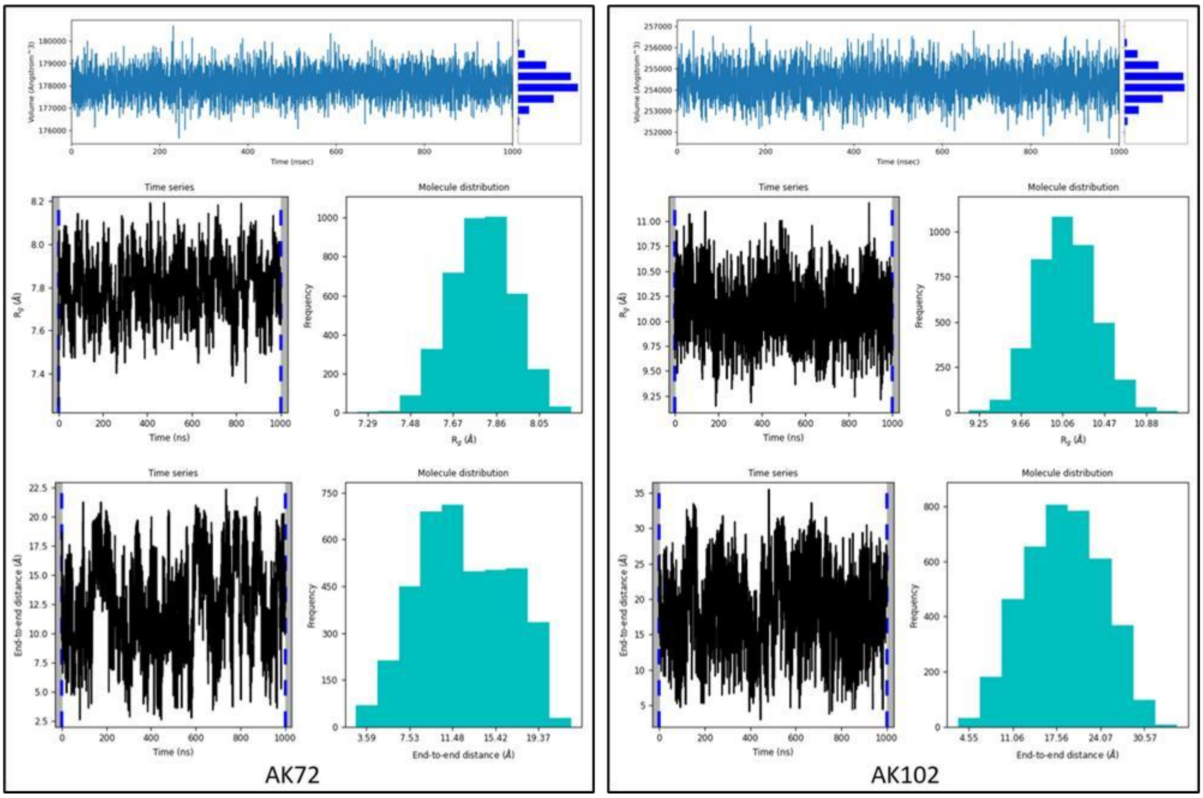

Figure S13: Statistics of MD simulations of three-dimensional models of siRNA, AK71, AK72, and AK102 for 1 µs at 300K and 1 atm. The four graphs show volume (Å^3), radius of gyration (Å), and longest end-to-end distance (Å) at top, middle, and bottom.

Title	# nonH atoms	MW [g/mol]	# Atoms in ring systems	# Bonds	# Bonds in ring systems	# ring systems	Ramification index	# 5-membered rings	# 6-membered rings	Cyclization degree	# rotors
AK35	102	1450.8	72	114	72	13	24	6	7	0.71	42
AK71	132	1859.5	114	150	114	19	36	0	19	0.86	36
AK72	120	1691.2	102	138	102	19	36	12	7	0.85	36

Table S1: Selected molecular descriptors of three-dimensional models of AK35, AK71, and AK72.

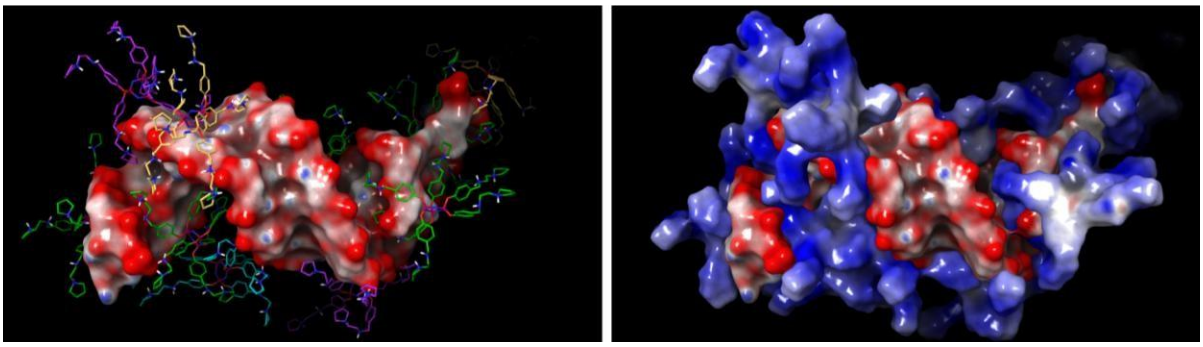

Figure S14: Final snapshot of AK35/siRNA dendriplex in 1:10 stoichiometry of 1 µs MD simulation. AK35 is depicted by tubes (left) or SASA (right), siRNA by SASA; no solvent molecules or ions are depicted for clarity. Left: only polar hydrogen atoms are shown; individual AK35 molecules are distinguished by various colors for carbon atoms. Color coding of nitrogen, oxygen, phosphor and hydrogen atoms and electrostatic potential as in publication Figure 3.

a)

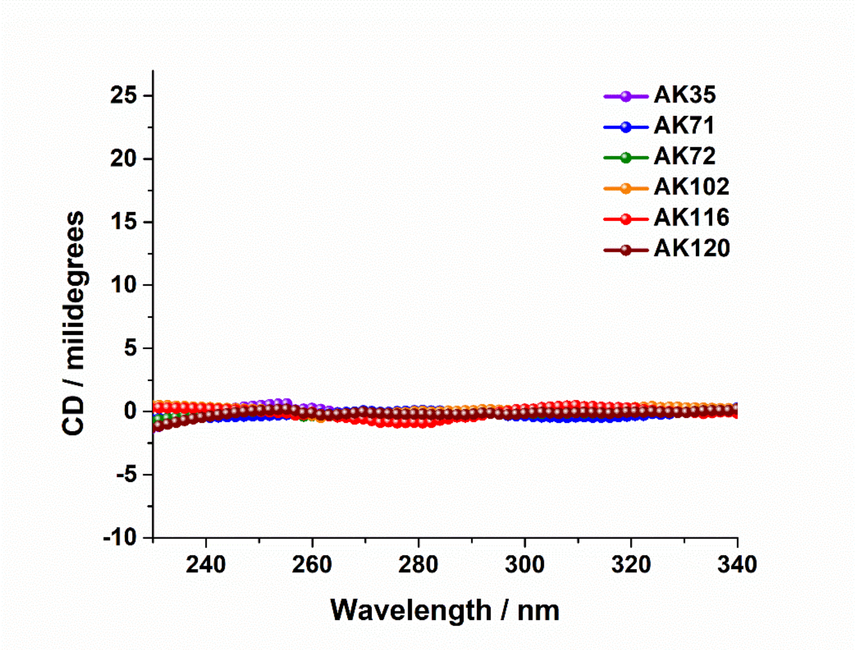

b)

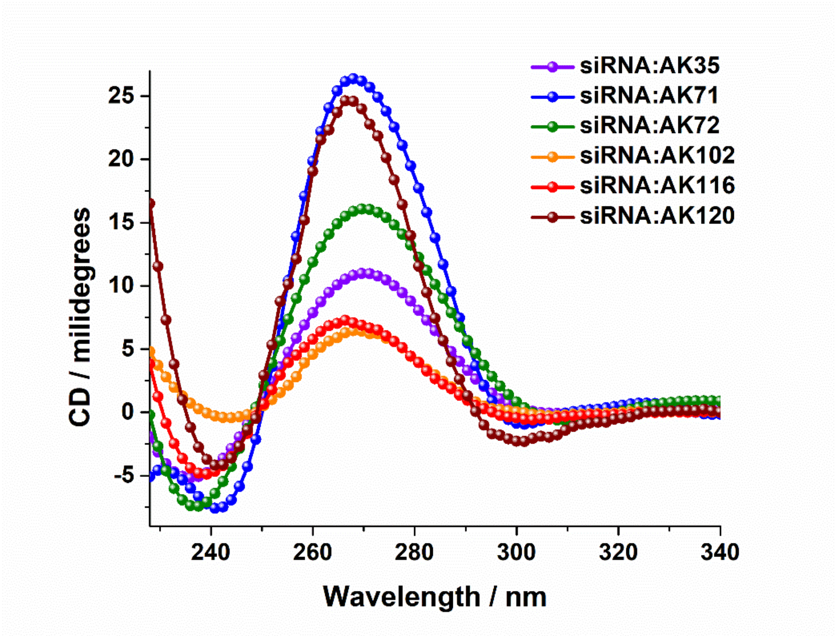

Figure S15. Circular dichroism (CD) spectra of (a) dendrimers and (b) their complexes with siRNA in water.

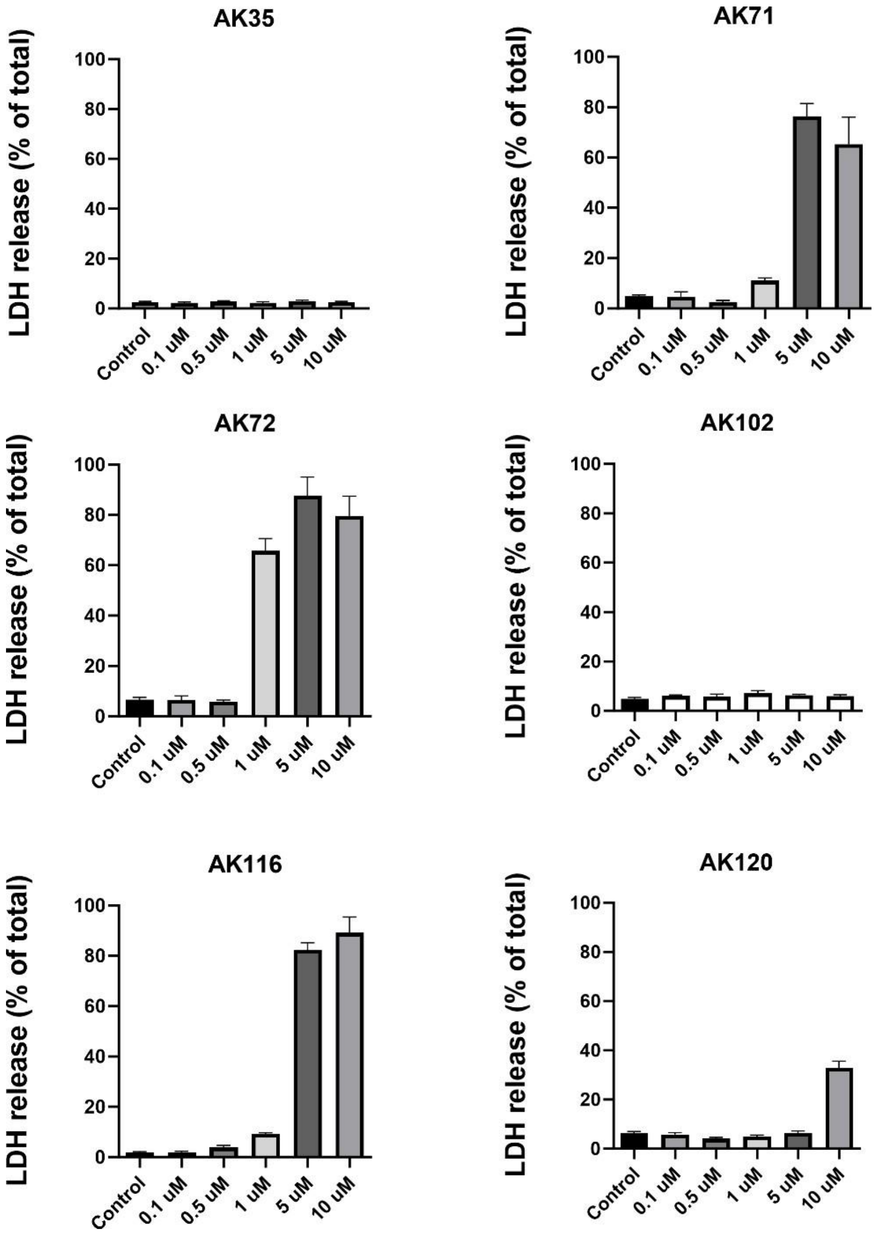

Figure S16. Toxicity of cationic phosphorus dendrimers on primary mouse astrocytes. Cells were exposed to the indicated dendrimer concentrations for 72 hours and toxicity was measured as LDH release to the culture medium as indicated in Methods. Data represent mean ± s.e.m of 8 to 12 experiments.

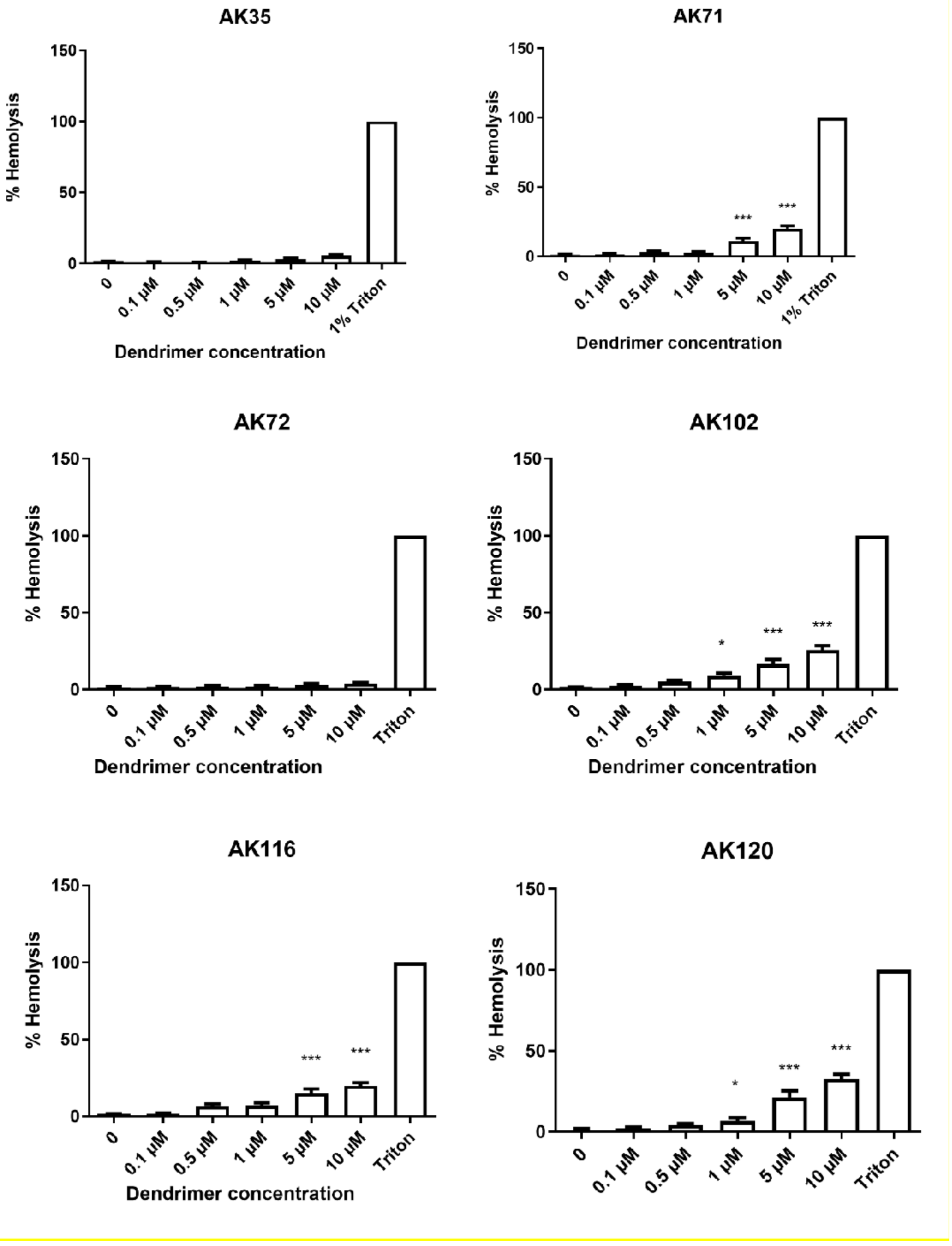

Figure S17. Hemolytic effect of phosphorus dendrimers. Red blood cells (RBCs) were exposed to increasing concentrations ranging from 0.1 to 10 µM) of phosphorus dendrimers and hemolysis was quantified as described in the Experimental section. 1% Triton was used as positive control (100%) lysing all the RBCs. Data represent mean ± s.e.m of 4 independent experiments. *p<0.05; ***p<0.01 when compared to control in absence of dendrimers

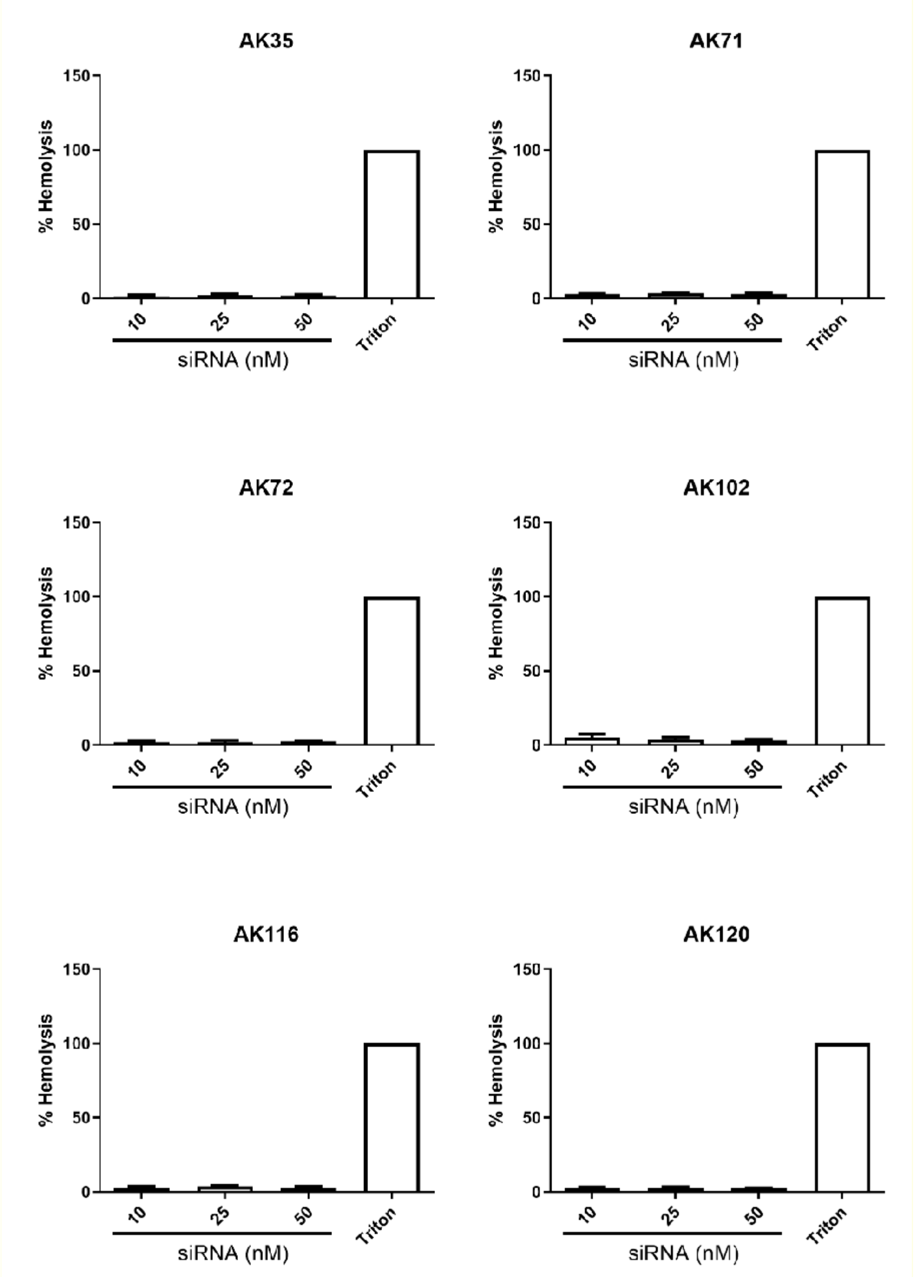

Figure S18. Hemolytic effect of dendriplexes. Red blood cells (RBCs) were exposed to dendriplexes formed by the following concentrations of phosphorus dendrimers: AK35 (10 µM), AK71 (1 µM), AK72 (10 µM), AK102 (1 µM), AK116 (1 µM), and AK120 (1 µM) plus 3 different scramble siRNA concentrations (10, 25 and 50 nM). Hemolysis was quantified as described in the Experimental section. 1% Triton was used as positive control (100%) lysing all the RBCs. Data represent mean ± s.e.m of 4 independent experiments.

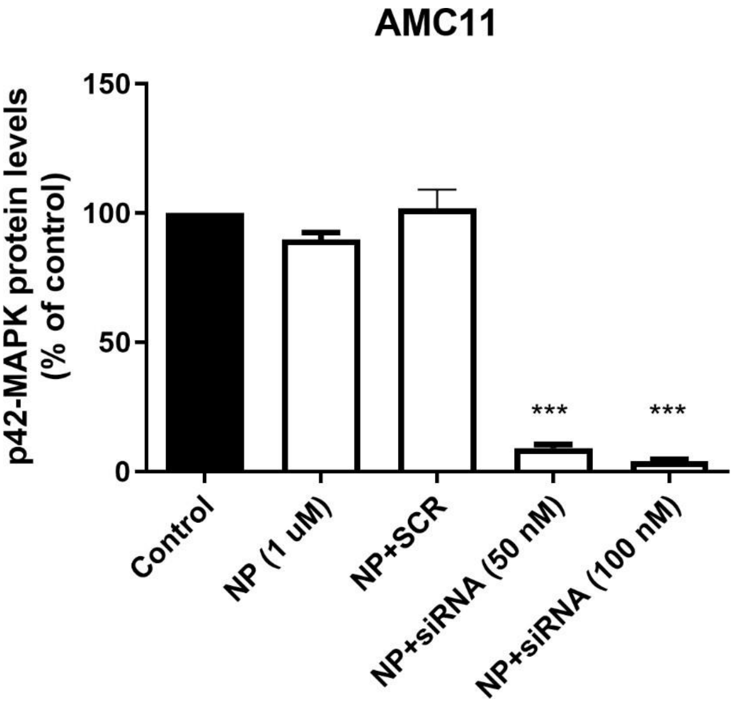

Figure S19. Transfection efficiency of the β-cyclodextrin derived nanoparticle AMC11 on p42-MAPK protein levels in T98G glioblastoma cells. T98G glioblastoma cells were incubated for 72 hours with dendriplexes containing AMC11 (one µM) and the indicated concentartions of specific siRNA directed against p42-MAPK mRNA or scramble siRNA (SCR). Cellular protein levels of p42-MAPK were detected by western blot as indicated in Methods. Data represent mean ± s.e.m. of 4 experiments. Control represents the protein levels in untreated cells taken as reference value (100 %).

6. References

[1] S. Mignani, V.D. Tripathi, D. Soam, R.P. Tripathi, S. Das, S. Singh, R. Gandikota, R. Laurent, A. Karpus, A.M. Caminade, A. Steinmetz, A. Dasgupta, K.K. Srivastava, J.P. Majoral, Safe Polycationic Dendrimers as Potent Oral In Vivo Inhibitors of Mycobacterium tuberculosis: A New Therapy to Take Down Tuberculosis, Biomacromolecules, **2021**, *22*, 2659-2675.

[2] Q. Vicens, E. Westhof, Crystal structure of paromomycin docked into the eubacterial ribosomal decoding A site, Structure, **2021**, *9*, 647-658.

[3] Q. Han, Q. Zhao, S. Fish, K.B. Simonsen, D. Vourloumis, J.M. Froelich, D. Wall, T. Hermann, Molecular recognition by glycoside pseudo base pairs and triples in an apramycin-RNA complex, Angew Chem Int Ed Engl, **2025**, *44*, 2694-2700.

[4] J. Kondo, B. Francois, A. Urzhumtsev, E. Westhof, Crystal structure of the Homo sapiens cytoplasmic ribosomal decoding site complexed with apramycin, Angew Chem Int Ed Engl, **2006**, *45*, 3310-3314.

[5] T. Hermann, V. Tereshko, E. Skripkin, D.J. Patel, Apramycin recognition by the human ribosomal decoding site, Blood Cells Mol Dis, **2007**, *38*, 193-198.

[6] M. Shalev, J. Kondo, D. Kopelyanskiy, C.L. Jaffe, N. Adir, T. Baasov, Identification of the molecular attributes required for aminoglycoside activity against Leishmania, Proc Natl Acad Sci U S A, **2013**, *110*, 13333-13338.

[7] A. Tsai, S. Uemura, M. Johansson, E.V. Puglisi, R.A. Marshall, C.E. Aitken, J. Korlach, M. Ehrenberg, J.D. Puglisi, The impact of aminoglycosides on the dynamics of translation elongation, Cell Rep, **2013**, *3*, 497-508.
